# C/EBPβ promotes poly(ADP-ribose) polymerase inhibitor resistance by enhancing homologous recombination repair in high-grade serous ovarian cancer

**DOI:** 10.1038/s41388-021-01788-4

**Published:** 2021-05-08

**Authors:** Jiahong Tan, Xu Zheng, Mengchen Li, Fei Ye, Chunyan Song, Cheng Xu, Xiaoxue Zhang, Wenqian Li, Ya Wang, Shaoqing Zeng, Huayi Li, Gang Chen, Xiaoyuan Huang, Ding Ma, Dan Liu, Qinglei Gao

**Affiliations:** 1 Cancer Biology Research Center (Key Laboratory of the Ministry of Education), Tongji Hospital, Tongji Medical College, Huazhong University of Science and Technology, Wuhan, People’s Republic of China; 2 Department of Obstetrics and Gynecology, Tongji Hospital, Tongji Medical College, Huazhong University of Science and Technology, Wuhan, People’s Republic of China; 3 Department of Neurosurgery, Tongji Hospital, Tongji Medical College, Huazhong University of Science and Technology, Wuhan, People’s Republic of China

**Keywords:** Cancer therapy, Gynaecological cancer, DNA damage and repair

## Abstract

PARP inhibitors (PARPi) are efficacious in treating high-grade serous ovarian cancer (HG-SOC) with homologous recombination (HR) deficiency. However, they exhibit suboptimal efficiency in HR-proficient cancers. Here, we found that the expression of CCAAT/enhancer-binding protein β (C/EBPβ), a transcription factor, was inversely correlated with PARPi sensitivity in vitro and in vivo, both in HR-proficient condition. High C/EBPβ expression enhanced PARPi tolerance; PARPi treatment in turn induced C/EBPβ expression. C/EBPβ directly targeted and upregulated multiple HR genes (BRCA1, BRIP1, BRIT1, and RAD51), thereby inducing restoration of HR capacity and mediating acquired PARPi resistance. C/EBPβ is a key regulator of the HR pathway and an indicator of PARPi responsiveness. Targeting C/EBPβ could induce HR deficiency and rescue PARPi sensitivity accordingly. Our findings indicate that HR-proficient patients may benefit from PARPi via targeting C/EBPβ, and C/EBPβ expression levels enable predicting and tracking PARPi responsiveness during treatment.

## Introduction

Epithelial ovarian cancer is the most lethal gynecological cancer, accounting for more than 95% of ovarian malignancies [1]. Annually, there are 230 000 new cases and 150 000 deaths worldwide [2]. High-grade serous ovarian cancer (HG-SOC) accounts for 75% of epithelial ovarian cancer, making it the most common histological type [2]. HG-SOC is characterized by genomic instability, with acquired or inherited mutations in different DNA repair pathways [1].

To maintain genomic stability, organisms have evolved DNA damage responses (DDRs) to cope with constant endogenous and environmental genotoxic insults [3]. Double-strand breaks (DSBs) are extremely cytotoxic and difficult to repair [4]. The homologous recombination (HR) pathway is preferentially adopted to repair DSBs [5]. HR-deficient cells cannot utilize HR to repair DSBs, rendering them vulnerable to poly(ADP-ribose) polymerase inhibitors (PARPi) [6, 7]. Genomic analyses have revealed that HR deficiency (HRD) contributes to ~50% of HG-SOC cases [6, 8]. Exposing these cells to PARPi will lead to DNA damage accumulation, cytotoxic genomic instability, and eventually synthetic lethality [9]. PARPi have been successfully implemented for recurrent HG-SOCs by leveraging inherent HRD [2].

Clinical trials have shown that patients with stable disease or partial response after platinum-based chemotherapy benefited from PARPi [1]. To date, olaparib, niraparib, and rucaparib have been approved for clinical application in platinum-sensitive recurrent HG-SOC [10]. PARPi show a range of efficacy on a continuum from patients with BRCA mutation, to those having HRD and even those without HRD [1, 10]. HR-deficient tumors, which have a “BRCAness profile”, show increased response to platinum and PARPi [1, 6]. PARPi show suboptimal efficiency toward HR-proficient tumors, which constitute a non-negligible proportion of all HG-SOCs, owing to the insufficiency in inducing synthetic lethality [6, 8, 11, 12]. Although PARPi activity extends beyond HRD, the exact distinctions between PARPi responders and non-responders remain unclear [6]. Currently, platinum sensitivity has been recognized as a functional indicator for PARPi application [13]. Tailoring precision medicine to individuals requires refining predictive biomarkers to help select patients who will benefit from PARPi [14].

Another issue related to PARPi application is the gradually emerging resistance [13, 14]. Despite the efficacy of PARPi, many patients inevitably develop acquired resistance because of factors such as restoration of the HR function, secondary mutations, drug efflux, and new mutations in other DDR genes [13, 14]. Approaches to eliminate PARPi resistance warrant further investigation.

We have previously found that CCAAT/enhancer-binding protein β (C/EBPβ) is an important determinant of BRCA1-related DDR [15]. C/EBPβ is a basic leucine zipper transcription factor and implicated in many cellular biological processes, such as cell proliferation, differentiation, apoptosis, oncogene-induced senescence, and tumorigenesis [16]. Knocking down C/EBPβ suppresses the expression of several genes in BRCA1-related DDR pathways [15]. Moreover, C/EBPβ enhances platinum resistance and helps predict platinum sensitivity [15]. Besides, C/EBPβ is an independent prognostic factor for HG-SOC [15]. Therefore, we speculated that C/EBPβ may promote PARPi resistance.

In this study, we have investigated the relationship between C/EBPβ expression and PARPi responsiveness. We found that C/EBPβ promotes PARPi resistance by directly targeting and upregulating multiple key genes in the HR pathway. Furthermore, C/EBPβ could be exploited as an indicator of PARPi responsiveness and a therapeutic target. Targeting C/EBPβ will rescue PARPi sensitivity and provide a therapeutic target for HR-proficient tumors, thereby maximizing the effectiveness of PARPi in treating ovarian cancer.

## Results

### C/EBPβ regulates DNA damage repair signals

**Fig. 1:**
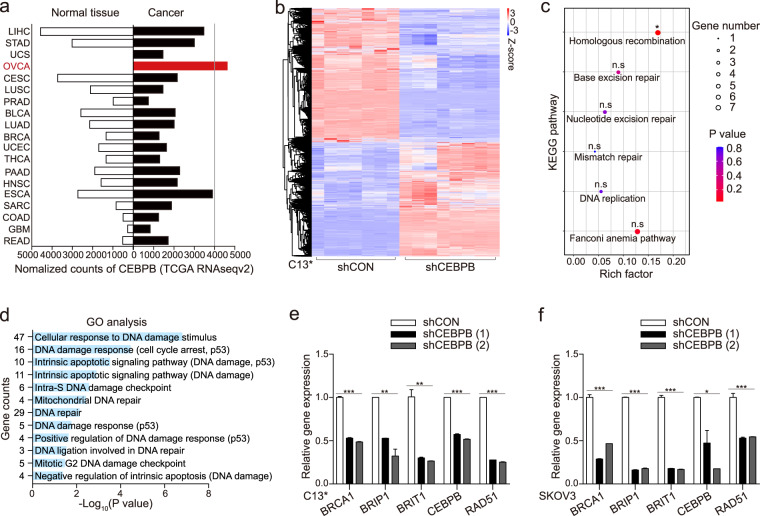
C/EBPβ regulates DNA damage repair signals. **a** TCGA RNAseqv2 data depicting CEBPB expression across a range of cancers. LIHC liver hepatocellular carcinoma, STAD stomach adenocarcinoma, UCS uterine carcinosarcoma, OVCA ovarian cancer, CESC cervical squamous cell carcinoma, LUSC lung squamous cell carcinoma, PRAD prostate adenocarcinoma, BLCA bladder urothelial carcinoma, LUAD lung adenocarcinoma, BRCA breast cancer, UCEC uterine corpus endometrial carcinoma, THCA thyroid carcinoma, PAAD pancreatic adenocarcinoma, HNSC head and neck squamous cell carcinoma, ESCA esophageal carcinoma, SARC sarcoma; COAD colon adenocarcinoma, GBM glioblastoma multiforme, READ rectum adenocarcinoma. RNA-sequencing of C13^*^ shCON (*N* = 7) and C13^*^ shCEBPB (*N* = 8) were performed. **b** Heatmap of differentially expressed genes (DEGs) after C/EBPβ knockdown in C13^*^. DEGs were subjected to enrichment analysis. **c** The effects of C/EBPβ knockdown on DDR-related KEGG pathways. **d** Enriched Gene Ontologies after C/EBPβ knockdown. The expression of the four HR genes, whose expression was significantly correlated with C/EBPβ expression, was detected in (**e**) C13^*^ and (**f**) SKOV3 after C/EBPβ manipulation. Each sample had triplicates and data were presented as mean ± SEM of three experiments (Student’s *t* test). *P* value was denoted as *P < 0.05, **P < 0.01, and ***P < 0.001, “n.s” represents “not significant”.

We had previously reported a significantly increased C/EBPβ expression in HG-SOC [15]. To further clarify the role of C/EBPβ in ovarian cancer, TCGA RNAseqv2 dataset was analyzed. Among all the tumors surveyed, ovarian cancer showed the highest C/EBPβ expression (Fig. 1a). RNA-sequencing of the HR-proficient cell line C13^*^ revealed that knocking down C/EBPβ changed its gene expression profile (Fig. 1b, Supplementary Table 1). Results of the RNA-sequencing were validated in two cancer cell lines (Supplementary Fig. S1a, S1b). Knockdown C/EBPβ significantly affected the KEGG pathway “Homologous recombination” (Fig. 1c, Supplementary Table 2). These differentially expressed genes (DEGs) also significantly accumulated in DDR-related Gene Ontologies (Fig. 1d; Supplementary Table 3). Therefore, we retrieved all pathways under “Replication and Repair” in the KEGG database, including ko03030 DNA replication, ko03410 Base excision repair, ko03420 Nucleotide excision repair, ko03430 Mismatch repair, ko03440 Homologous recombination, ko03450 Non-homologous end-joining, and ko03460 Fanconi anemia pathway, and obtained a total of 210 non-redundant genes. There was a significant overlap between the DEGs after C/EBPβ knockdown and these retrieved DDR genes (*P* < 0.0001; Supplementary Fig. S1c). Depletion of C/EBPβ altered the expression profile of these 97 overlapping genes (Supplementary Fig. S1d). Consistent with enrichment analyses, the expression levels of four DDR genes, namely BRCA1, BRIP1, BRIT1, and RAD51, significantly correlated with C/EBPβ expression in the TCGA dataset, although some of them had no statistically significant expression difference (Supplementary Fig. S2). These four DDR genes were also downregulated after C/EBPβ depletion in C13^*^ (Fig. 1e) and SKOV3 (Fig. 1f). These findings indicated that C/EBPβ regulated DDR signals in ovarian cancer.

### High C/EBPβ expression promotes PARPi resistance

**Fig. 2:**
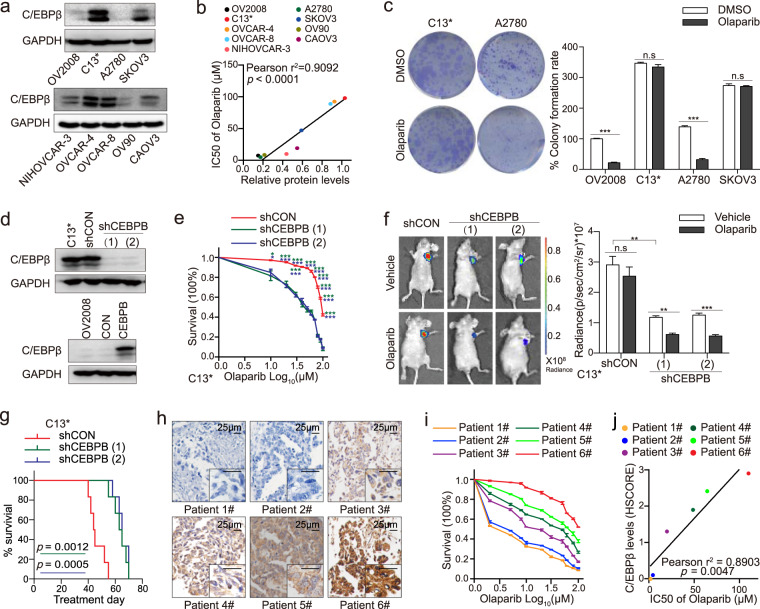
High C/EBPβ expression promotes PARPi resistance. **a** Western blotting analysis detected C/EBPβ expression in cancer cell lines. The experiment was repeated thrice and representative images were shown. **b** Correlation analysis of C/EBPβ expression levels and IC50s of cancer cell lines for olaparib (Pearson’s correlation test). **c** Colony-formation rates of cancer cell lines under olaparib exposure. Each assay was conducted in triplicates and error bars indicated mean ± SEM of three assays (Student’s *t* test). Lentiviruses were used to modulate C/EBPβ expression. **d** After transfection, C/EBPβ expression was detected using western blot. A representative blot of three assays was shown. **e** Cell viability assays for C/EBPβ-depleted C13^*^ cells (Student’s *t* test). Each sample had six replicates and all experiments had triplicates. Error bars represented mean ± SEM. Stably transfected C13^*^ cells were subcutaneously inoculated into mouse flanks. Olaparib or vehicle was administered (six mice per group). **f** Tumor sizes were monitored using in vivo bioluminescent imaging, and tumor burdens were quantified by total radiance. Representative images of mouse xenografts were shown (Student’s *t* test). Error bar, mean ± SEM. **g** Kaplan–Meier survival plot of mice after C/EBPβ manipulation and olaparib treatment (Log-rank test). Freshly collected specimens were used for primary cultures. **h** Immunohistochemical analysis of C/EBPβ expression in tumor tissues. **i** Cell viability assays of primary cultures. Each assay was conducted in six wells and repeated three times. Error bars depicted mean ± SEM. **j** Correlation analysis of C/EBPβ expression levels and IC50s of primary cultures for olaparib (Pearson’s correlation test). *P* value was denoted as * *P* < 0.05, ** *P* < 0.01, and *** *P* < 0.001, “n.s” represents “not significant”.

Accordingly, C/EBPβ might affect PARPi responsiveness. By exploring DepMap, we found that olaparib resistance of HR-proficient cancer cell lines positively correlated with C/EBPβ expression (Supplementary Fig. S3). Endogenous C/EBPβ levels were significantly correlated with olaparib resistance in vitro (Fig. 2a, b; Supplementary Fig. S4a). Cells having low C/EBPβ expression, such as OV2008 and A2780, showed low viability even at a small dose of olaparib, while C13^*^, which exhibited strong C/EBPβ expression, survived under an increased olaparib concentration. Similar results were obtained using another two PARPi AZD2461 and BMN673 (Supplementary Fig. S4b, c). Compared with OV2008 and A2780, C13^*^ exhibited increased cell viability when treated with AZD2461 and BMN673. Under olaparib exposure, A2780 formed significantly lesser and smaller colonies, whereas the clonogenicity of C13^*^ was only slightly affected (Fig. 2c).

To further confirm these observations, we constructed C/EBPβ knock-in and knock-out cell lines using OV2008 and C13^*^ (Fig. 2d) [17]. C/EBPβ knockdown sensitized C13^*^ to olaparib, and C/EBPβ overexpression enhanced cell viability under olaparib exposure (Fig. 2e, Supplementary Fig. S4d). Similar results were observed in colony formation assays (Supplementary Fig. S4e, f). The effectiveness of olaparib treatment was verified using western blotting analysis of PAR (Supplementary Fig. S4g). The expression of C/EBPβ and cleaved caspase3 varied in the opposite direction further supported that C/EBPβ promoted PARPi resistance (Supplementary Fig. S4h). Stably transfected C13^*^ or OV2008 cells were subcutaneously inoculated into mouse flanks. At the indicated time, tumor sizes were monitored using in vivo bioluminescent imaging. High C/EBPβ expression conferred olaparib tolerance in vivo. Tumor growth was only slightly suppressed by olaparib in mice engrafted with C13^*^ shCON cells, while mice inoculated with C13^*^ shCEBPB cells exhibited decreased luciferase expression and were sensitive to olaparib (Fig. 2f). Xenografts models of OV2008 CON cells showed sensitivity toward olaparib, wherein tumors were restrained and even eliminated, while the C/EBPβ-overexpressing group was insensitive to olaparib and had heavier tumor burdens (Supplementary Fig. S4i). We also examined the effect of C/EBPβ on survival, with survival cut-off criteria defined as tumor volume reaching 1500 mm^3^ or an observation duration of 90 days. C/EBPβ depletion substantially improved survival compared with that of the control (Fig. 2g), while C/EBPβ overexpression deteriorated survival outcome (Supplementary Fig. S4j). C/EBPβ expression was also interfered with siRNA in OV2008 and A2780 cells. Depletion of C/EBPβ did not further enhance PARPi responsiveness (Supplementary Fig. S5a–S5d).

The relationship between C/EBPβ expression and olaparib sensitivity was also examined in six primary cultures. After confirmation of epithelial content (Supplementary Fig. S5e), cell viability assays were performed. Cell survival upon olaparib treatment was directly proportional to C/EBPβ expression (Fig. 2h, i; Supplementary Fig. S5f). Correlation analysis showed that olaparib resistance was positively related to C/EBPβ expression (*r*^2^ = 0.8903, *P* = 0.0047; Fig. 2j). The negative relation between C/EBPβ and cleaved caspase3 was also observed (Supplementary Fig. S5g, h), but the relation between C/EBPβ and Ki-67 in the six patients was not significant (Supplementary Fig. S5i). To explore the influences of C/EBPβ on apoptosis, expression of cleaved caspase8 and cleaved PARP1 was also examined (Supplementary Fig. S6a, b). Depletion of C/EBPβ exacerbated apoptosis in C13^*^ cells, which could be rescued by adding the caspase inhibitor Q-VD-OPh, while overexpressed C/EBPβ attenuated apoptosis in OV2008 cells (Supplementary Fig. S6c, d). C/EBPβ also affected ROS levels in C13^*^ and OV2008 cells and regulated the expression of reductases GSTP1 and NQO1 (Supplementary Fig. S7). Taken together, C/EBPβ expression was inversely correlated with PARPi sensitivity.

### Olaparib treatment induces C/EBPβ expression

**Fig. 3:**
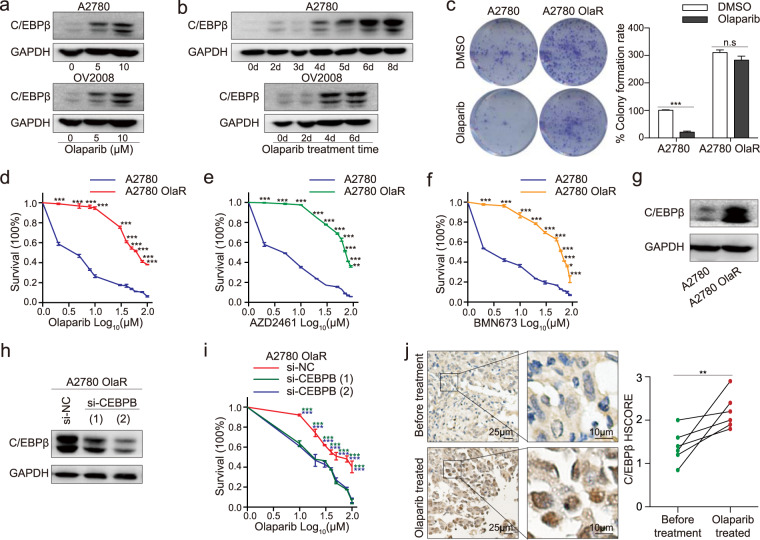
Olaparib treatment induces C/EBPβ expression. A2780 and OV2008 were exposed to olaparib for (**a**) different concentrations and (**b**) different periods. Subsequently, C/EBPβ expression was detected by western blot and the experiments were repeated three times. A2780 was exposed to olaparib to generate the resistant strain A2780 OlaR. **c** Colony-formation rates of A2780 and A2780 OlaR under olaparib exposure. Each sample had triplicate wells and data represented mean ± SEM of three assays (Student’s *t* test). Cell viability assays for A2780 and A2780 OlaR under different concentrations of (**d**) olaparib, (**e**) AZD2461, and (**f**) BMN673. Each sample had six replicates and error bars were mean ± SEM of three experiments (Student’s *t* test). **g** C/EBPβ expression in A2780 OlaR was detected by western blot and repeated three times. **h** siRNA interference of C/EBPβ expression was used in A2780 OlaR. Western blotting analysis was performed and repeated thrice. **i** Cell viability assays for A2780 OlaR after CEBPB interference. The experiment was repeated for thrice in six replicates for each cell line and error bars depicted mean ± SEM (Student’s *t* test). **j** Immunohistochemical staining of C/EBPβ in paired specimens before and after olaparib treatment (Student’s *t* test). *P* value was denoted as *P < 0.05, **P < 0.01, and ***P < 0.001, “n.s” represents “not significant”.

To clarify the reciprocal relationship between C/EBPβ and PARPi responsiveness, we treated A2780 and OV2008 cells with olaparib at different concentrations and for different periods. Surprisingly, Olaparib induced C/EBPβ expression in a dose-dependent manner and also in a time-dependent manner (Fig. 3a, b; Supplementary Fig. S8a, b).

A stable resistant cell strain was generated by culturing A2780 in the continued presence of olaparib. Resistance emerged with a gradual increase in exposure. When the IC50 of the resistant strain was ≥30 times that of the parent line, an olaparib-resistant strain had then been developed and referred to as A2780 OlaR. When exposed to the same concentration of olaparib, A2780 OlaR formed more colonies and had less apoptosis (Fig. 3c, Supplementary Fig. S8c). By cell survival assays, we obtained similar results (Fig. 3d). Consistently, A2780 OlaR expressed less cleaved capsase3 than A2780 (Supplementary Fig. S8d). We had also ceased olaparib treatment for one month, which had a hardly measurable influence on the drug tolerance of A2780 OlaR (Supplementary Fig. S8e). Cell cycle analysis showed subtle changes between A2780 and A2780 OlaR, implying that the alteration of drug responsiveness was independent of the cell cycle (Supplementary Fig. S8f). Concurrently, resistance was maintained when A2780 OlaR was treated with AZD2461 or BMN673 (Fig. 3e, f).

We then examined C/EBPβ expression in A2780 OlaR and observed a pronounced increase in C/EBPβ expression (Fig. 3g, Supplementary Fig. S8g). C/EBPβ elimination by siRNA interference rendered A2780 OlaR sensitive to olaparib without affecting cell viability (Fig. 3h, i; Supplementary Fig. S8h).

Clinical specimens obtained from patients who had HR-proficient HG-SOC and had undergone several rounds of olaparib treatment were used to further explore the mutual relationship between C/EBPβ and PARPi. We found increased C/EBPβ expression after olaparib treatment (Fig. 3j), along with decreased cleaved caspase3 expression (Supplementary Fig. S9). The effect of C/EBPβ on PARPi responsiveness was also observed in the HR-proficient breast cancer cell line MDA-MB-231, but not in the BRCA1-mutant MDA-MB-436 (Supplementary Figs. S10, S11). In summary, PARPi administration induced C/EBPβ expression, and C/EBPβ in turn promoted PARPi resistance.

### C/EBPβ directly targets HR genes and affects DNA damage repair

**Fig. 4:**
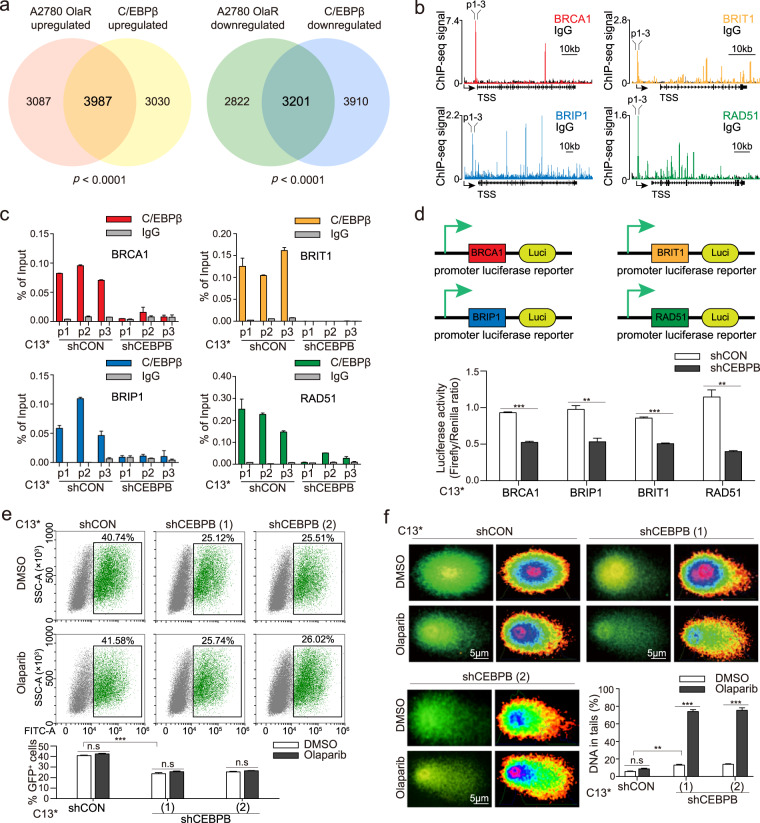
C/EBPβ directly targets HR genes and affects DNA damage repair. **a** RNA-sequencing of A2780 (*N* = 8) and A2780 OlaR (*N* = 8) was performed, and the DEGs overlapped with the DEGs identified after C/EBPβ knockdown (Chi-squared test). **b** Prediction of C/EBPβ-binding affinity using Cistrome DB. Chromatin immunoprecipitation was performed in triplicates with anti-C/EBPβ antibodies in C13^*^ shCON and shCEBPB cells and the experiments were repeated twice. **c** The binding ability of C/EBPβ for HR genes was detected using ChIP-qPCR. ChIP-qPCR was performed in triplicate wells and primers used were designed at the sites indicated in **b**. Error bar, mean ± SEM. **d** Promoter luciferase reporter assays of the four HR genes in C13^*^ shCON and shCEBPB cells. The experiments were conducted in triplicates and repeated three times. Data were presented as mean ± SEM (Student’s *t* test). **e** HR reporter assays in C13^*^ shCON and shCEBPB cells after olaparib treatment. Each sample had triplicates and error bars represented mean ± SEM of three experiments (Student’s *t* test). **f** Comet assays with C13^*^ shCON and shCEBPB cells after olaparib treatment. Results were obtained from three independent experiments and depicted as mean ± SEM (Student’s *t* test). *P* value was denoted as *P < 0.05, **P < 0.01, and ***P < 0.001, “n.s” represents “not significant”.

To determine the mechanisms underlying the effects of C/EBPβ on PARPi resistance, we performed RNA-sequencing of A2780 OlaR and its parent line A2780 (Supplementary Fig. S12a, b; Supplementary Table 4). Surprisingly, these DEGs significantly overlapped with the DEGs regulated by C/EBPβ (*p* < 0.0001; Fig. 4a). These results implied that C/EBPβ was strongly correlated with the transformation from sensitivity to resistance.

Since C/EBPβ is a transcription factor, we checked the functional categories of C/EBPβ targets by reanalyzing the chromatin immunoprecipitation sequencing (ChIP-seq) data [15]. We found that 20% of HR genes were C/EBPβ targets (Supplementary Fig. S12c). The binding affinity of C/EBPβ for the HR genes was then predicted in Cistrome DB. Four HR genes, namely BRCA1, BRIP1, BRIT1, and RAD51, were found to be potential targets of C/EBPβ (Fig. 4b). All four genes scored high at both C/EBPβ motifs when validated in JASPAR (Supplementary Fig. S12d, Supplementary Table 5). This finding was further confirmed by ChIP-qPCR, where the high binding affinity of C/EBPβ for these four genes was significantly abolished after C/EBPβ knockdown (Fig. 4c). Promoter luciferase reporter plasmids for these four HR genes were constructed. Depletion of C/EBPβ significantly repressed the luciferase activity of reporter plasmids (Fig. 4d). These results indicated that C/EBPβ directly targeted these four HR genes.

To ascertain the effects of C/EBPβ on HR-mediated repair of DNA damage, HR reporter assays were performed. C/EBPβ knockdown reduced the ratio of GFP^+^ cells, while overexpressed C/EBPβ enhanced HR efficiency as indicated by more GFP^+^ cells (Fig. 4e; Supplementary Fig. S12e, S12f). To evaluate DNA damage, comet assay and γH2AX staining were performed [18]. Olaparib severely impaired DNA and induced more DNA in tails after C/EBPβ knockdown, while C/EBPβ overexpression helped maintain DNA integrity and diminished DNA in tails (Fig. 4f, Supplementary Fig. S13a). C/EBPβ depletion triggered γH2AX foci formation in C13^*^ cells and accumulated DNA damage, whereas exogenous augmentation of C/EBPβ in OV2008 cells inhibited γH2AX foci formation and protected cells from DNA damage (Supplementary Fig. S13b, c). Together, C/EBPβ directly targeted four HR genes and protected cells from DNA damage, thereby mediating PARPi resistance.

### C/EBPβ upregulates HR genes and induces HR restoration

**Fig. 5:**
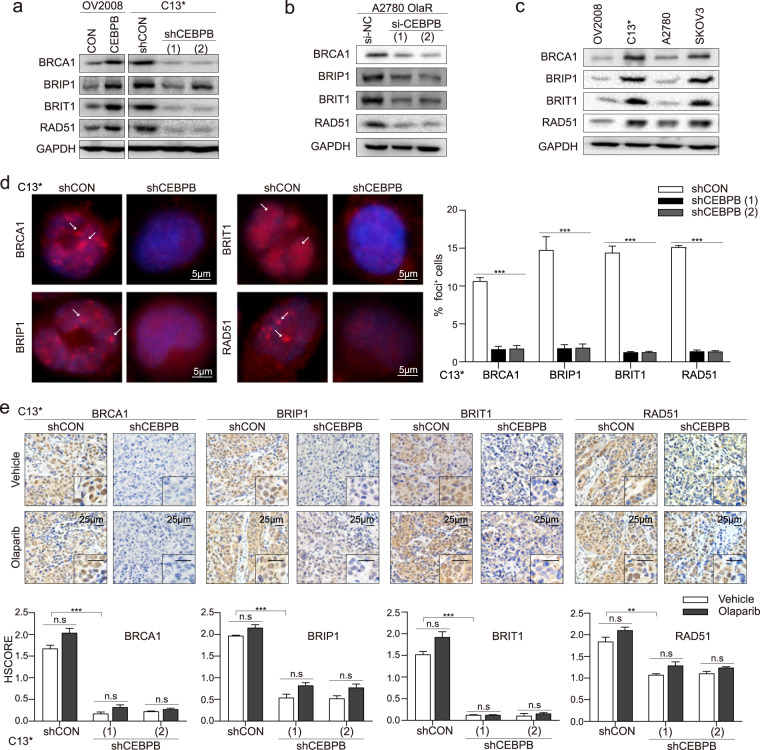
C/EBPβ upregulates HR genes and induces HR restoration. **a** HR genes expression was detected by western blot after C/EBPβ manipulation in OV2008 and C13^*^[35]. **b** HR genes expression in A2780 OlaR was detected using western blot after C/EBPβ knockdown. **c** HR genes expression in the four mainly used cancer cell lines was detected by western blot. By western blotting analysis, all experiments were repeated three times. **d** Immunofluorescence staining of HR proteins; representatives were shown (left). Staining foci were indicated by arrowheads. HR protein foci formation rates in C13^*^ shCON and shCEBPB cells after olaparib treatment were calculated (right). Data were obtained from three independent experiments and denoted as mean ± SEM (Student’s *t* test). **e** Tumor tissues from C13^*^ mouse models (six mice per group) were stained with the indicated antibody, and HR proteins expression was evaluated using HSCORE. Data shown represented mean ± SEM (Student’s *t* test). *P* value was denoted as *P < 0.05, **P < 0.01, and ***P < 0.001, “n.s” represents “not significant”.

HR gene panel perturbations after C/EBPβ manipulation were assessed. C/EBPβ knock-in upregulated HR genes expression in OV2008, while C/EBPβ knock-out reduced their expression in C13^*^ (Fig. 5a, Supplementary Fig. S14a, b). In A2780 OlaR, C/EBPβ-targeted siRNA interference decreased the expression of all four genes (Fig. 5b, Supplementary Fig. S14c). Similar results were noted in cancer cell lines, where HR genes expression varied along with C/EBPβ expression (Fig. 5c). Formation of HR proteins foci serves as an indicator of HR capacity [19]. Under olaparib exposure, C/EBPβ depletion suppressed HR proteins foci formation in C13^*^ (Fig. 5d), while C/EBPβ overexpression elevated their intranuclear foci formation rates in OV2008 (Supplementary Fig. S14d).

The effects of C/EBPβ on the HR pathway were maintained in vivo. C13^*^ and OV2008 mouse models were treated with 30 mg/Kg olaparib/vehicle intraperitoneally every 2 days for 40 days and the mice were sacrificed for tissue harvest after bioluminescent imaging. Immunohistochemical analyses of the four HR genes recapitulated our in vitro experiments. C/EBPβ depletion inhibited their expression, whereas their expression was enhanced by C/EBPβ overexpression (Fig. 5e; Supplementary Figs. S14e, f, and S15a). The same tendency was observed at their mRNA expression levels (Supplementary Fig. S15b, c). Similarly, cleaved caspase3 changed opposite to C/EBPβ, but no significant variation of Ki-67 expression was observed (Supplementary Fig. S15d–g). Overall, C/EBPβ upregulated HR genes and induced recovery of HR capacity.

### C/EBPβ promotes PARPi resistance through the HR pathway

**Fig. 6:**
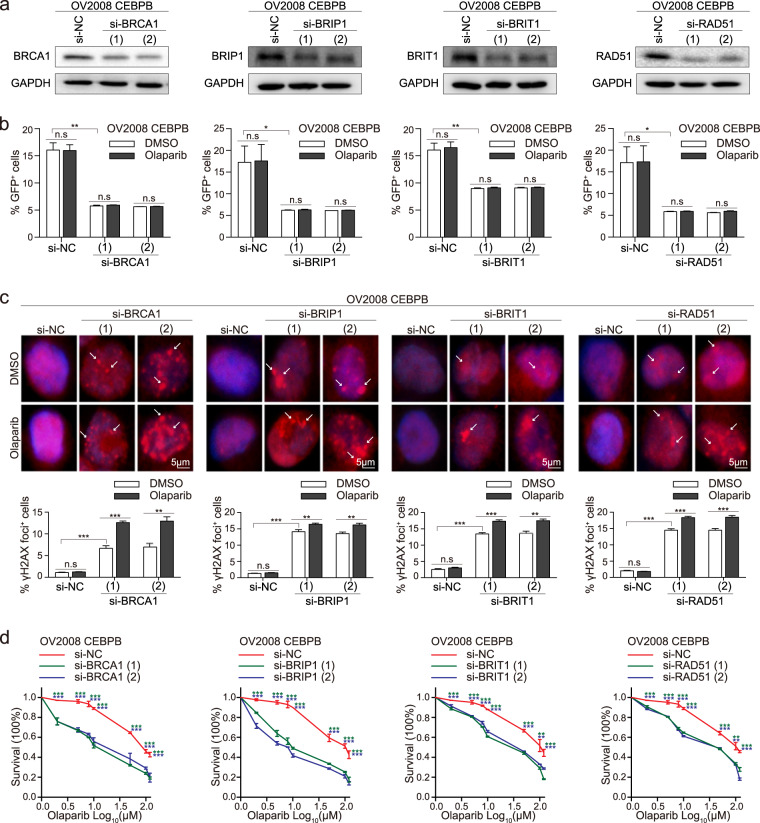
siRNA interference of HR genes abolishes the effects of C/EBPβ. C/EBPβ-overexpressing OV2008 cells were transfected with siRNA targeting BRCA1, BRIP1, BRIT1, or RAD51. **a** HR protein expression was detected by western blot after siRNA interference and repeated thrice. **b** HR reporter assays were performed after siRNA interference and olaparib treatment. Each sample had triplicates and the data represented mean ± SEM of three experiments (Student’s *t* test). **c** Immunofluorescence staining of γH2AX; representatives were shown (top). Staining foci were indicated by arrowheads. γH2AX foci formation rates were calculated after confirmation of transfection and olaparib treatment (bottom). Results were obtained from three independent experiments and presented as mean ± SEM (Student’s *t* test). **d** Cell viability assays under olaparib exposure after siRNA interference. The experiments were conducted thrice with six replicate wells. Data were shown as mean ± SEM (Student’s *t* test). *P* value was denoted as *P < 0.05, **P < 0.01, and ***P < 0.001, “n.s” represents “not significant”.

To rescue PARPi sensitivity in C/EBPβ-overexpressing cells, the expression of the four HR genes were depleted. siRNA interference of the four HR genes decreased the ratio of GFP^+^ cells in OV2008 CEBPB cells, indicating reduced HR efficiency (Fig. 6a, b). Similar results were observed in A2780 OlaR after siRNA interference (Supplementary Fig. S16a, b). Silencing of the four HR genes attenuated RAD51 foci formation rates in OV2008 CEBPB and A2780 OlaR (Supplementary Fig. S16c, d). γH2AX staining showed pronounced foci formation rate after siRNA transfection in OV2008 CEBPB cells (Fig. 6c). Consistent results were obtained in A2780 OlaR (Supplementary Fig. 17a). Cell viability assays showed that depletion of any of these four HR genes would enhance olaparib sensitivity (Fig. 6d, Supplementary Fig. S17b). Collectively, the findings indicated that C/EBPβ promoted PARPi resistance through the HR pathway.

## Discussion

Exploitation of dysregulated DDR is a highly promising approach to develop anti-cancer treatment [20]. C/EBPβ, a basic leucine zipper transcription factor, was found to affect BRCA1-related DDR [15]. In this study, we explored the reciprocal relationship between C/EBPβ and PARPi responsiveness. The results herein showed that C/EBPβ affected DDR signals, especially the HR pathway of ovarian cancer. C/EBPβ directly targeted and upregulated multiple HR genes, including BRCA1, BRIP1, BRIT1, and RAD51, inducing restoration of HR capacity and promoting PARPi resistance. Meanwhile, olaparib treatment induced C/EBPβ expression, implying that C/EBPβ contributed to acquired PARPi resistance. Thus, C/EBPβ could be exploited as an indicator of PARPi responsiveness and as a therapeutic target. Targeting C/EBPβ could induce HRD and rescue PARPi sensitivity accordingly.

HR, an error-free form of repair with high fidelity, is crucial for cell survival [5, 21]. C/EBPβ directly targeted and upregulated four key HR genes. BRCA1 plays a role in transcription, DNA repair of DSBs, and recombination. BRIP1 interacts with BRCA1 and the bound complex is important in the normal repair function of BRCA1. BRIT1 is a DDR protein and engages in G2/M checkpoint arrest. RAD51 functions in homologous pairing and strand transfer of DNA. C/EBPβ knockdown decreased the expression of these genes and reduced HR capacity. The effects of C/EBPβ on HR genes may not be confined to transcription regulation, which might further reinforce its potential. Besides, some other important HR genes, like BABAM1, were downregulated by C/EBPβ and involved in the transition from PARPi sensitivity to resistance. Therefore, C/EBPβ is potentially an important regulator of the HR pathway, providing a rationale for targeting C/EBPβ to induce HRD in HR-proficient tumors. Except for the HR pathway, the functional categories of C/EBPβ targets involve several DNA repair pathways [15]. To exclude the influences of mismatch repair (MMR), MMR-proficient cell lines (C13^*^, OV2008, and A2780) and MMR-deficient SKOV3 were used in this study [22–24]. Since there might be compensatory upregulation of other DNA repair pathways upon hypofunction of the HR pathway, C/EBPβ depletion cannot make the HR-proficient cancer cells as sensitive as the BRCA-mutated cells. However, the enhanced sensitivity deserves further exploitation.

PARPi, which specifically kill cancer cells with inadequate HR capacity, have benefited many HG-SOC patients [2, 10]. The emergence of resistance has precluded their further application; 40–70% of patients could not respond as expected to olaparib because of drug resistance [13, 14, 25]. In-depth research has been performed on specific predispositions to PARPi resistance [26–30]. Combination therapies were explored, but their uses were hindered by sequential orders and dose-limiting toxicity [31–35]. In this study, we used three PARPi: olaparib, AZD2461, and BMN673. Olaparib inhibits PARP1/2; AZD2461 is a poor substrate of p-glycoprotein and inhibits PARP1/2/3; BMN673 suppresses PARP1/2/3/4 and has the strongest PARP trapping ability [36–39]. All three PARPi could not trigger sufficient efficiency in C/EBPβ-overexpressing cells. Patients with endogenous overexpressed C/EBPβ were inherently resistant to PARPi. ROS was reported to have a role in PARPi responsiveness [40]. C/EBPβ could augment the expression of reductases to neutralize ROS levels and affect ROS distribution, which also contributed to olaparib tolerance. Besides, the effect of C/EBPβ was also observed in breast cancer cell line MDA-MB-231. Although there was no overt effect of C/EBPβ in the BRCA1 mutant MDA-MB-436, which was sensitive to PARPi. These findings indicate that C/EBPβ is an important and general mediator of PARPi resistance. Given that both platinum- and PARPi-based therapies function through affecting DNA repair machinery, there is cross-resistance between platinum and PARPi [2, 10, 41]. Platinum-resistant HG-SOC is not susceptible to PARPi [1, 2]. C/EBPβ has been previously found to enhance platinum resistance; C13^*^ and OV2008 represents cisplatin-resistant and cisplatin-sensitive cell line pair [15]. C/EBPβ knockdown could promote olaparib sensitivity in C13^*^. Therefore, targeting C/EBPβ also helps overcome this cross-resistance.

Predictive biomarkers for PARPi response are urgently required [14]. Although platinum sensitivity may provide a feasible indication of PARPi response, a few patients who did not respond to platinum reagents benefited from PARPi [1, 13]. Detection of HRD (especially BRCA1/2 mutations) remains the most convincing predictor of PARPi responsiveness [13, 38]. However, some HR-deficient patients were insensitive, while some HR-proficient patients benefited from PARPi [13, 14]. RAD51 detection is being considered as a promising approach, but its efficiency is restricted by exogenous stresses such as irradiation [13]. C/EBPβ directly modulated the HR pathway and targeted four key HR genes. In addition, C/EBPβ expression predicts platinum sensitivity and HG-SOC prognosis [15]. The positive relationship between C/EBPβ expression and olaparib resistance was successively validated in HR-proficient ovarian cancer cell lines, primary cultures of HG-SOC cancer tissues, and paired samples from HRD-negative patients. Excluding patients 1# and 2#, who were identified to have BRCA1 mutation lately, the effects of C/EBPβ on PARPi resistance were not affected. Thus, C/EBPβ detection holds great potential in predicting PARPi responsiveness; however, a large preclinical study is required for further confirmation. Furthermore, C/EBPβ detection using immunohistochemistry might be more cost-effective than gene screening in predicting and tracking PARPi responsiveness during treatment.

C/EBPβ, a potential therapeutic target in epithelial cancers, was reported to control cell proliferation and apoptosis [16]. The findings herein indicate that C/EBPβ inhibits apoptosis. Overexpressed C/EBPβ could suppress caspase-dependent induction of apoptosis. Reduced apoptosis in C/EBPβ-overexpressing cells contributed to C/EBPβ-mediated PARPi resistance. However, we could not determine the effect of C/EBPβ on proliferation. The number of clinical specimens and animal models was limited, and these factors could confound the results. Work is ongoing in a large cohort to determine whether C/EBPβ affects cell proliferation in HG-SOC. The underlying mechanisms of C/EBPβ upregulation upon PARPi treatment were complicated. There was the possibility that, under the selection pressure of PARPi, cells with higher endogenous C/EBPβ expression survived, leading to C/EBPβ upregulation upon PARPi treatment. Besides, the C/EBPβ-mediated apoptosis inhibition largely selected cells with overexpressed C/EBPβ for survival, which might also partially explain the phenomenon. C/EBPβ was found to be involved in epigenetic reprogramming [15]. The histone demethylase JMJD3 could escalate C/EBPβ expression [42]. Insulin and the PI3K/AKT axis were also reported to be implicated in C/EBPβ upregulation [43]. The potential mechanisms of PARPi-induced C/EBPβ elevation in ovarian cancer remain to be elucidated.

Owing to the significant heterogeneity, using appropriate cell lines to study HG-SOC is challenging [28]. Cell lines are extensively involved in preclinical research. However, we must acknowledge the insufficiency of this study. Based on genomic profiles, A2780 and SKOV3 were reported as unlikely HG-SOC cell lines [44]. A recent study, which integrated genomic, epigenomic, and expression analysis, has proposed that using these well-characterized cell lines during preclinical studies will provide translational rationales for anti-cancer treatment [45]. SKOV3 was revised as a possible HG-SOC cell line since there is a frameshift TP53 mutation [45]. Given the origin of C13^*^ and OV2008, their usage is somewhat indecent [46]. Although the primarily used cell line models had some controversies, the reciprocal relationship between C/EBPβ and PARPi responsiveness was confirmed in several likely/possible HG-SOC cell lines and primary cultures. Deriving cell lines from primary ovarian cancers could minimize the discrepancy between cell line models and clinical tumors [44]. Besides, in the current study, we focused on the HR pathway. However, the relationship between C/EBPβ and replication fork protection and other DDR pathways remains unclear. Although there are difficulties to specifically directly target C/EBPβ [16], Withaferin A might potentially suppress C/EBPβ [47, 48], JMJD3 targets and upregulates C/EBPβ expression in leukemia [42], and JAK-STAT3 signaling can activate C/EBPβ [49]. Withaferin A and inhibitors of JMJD3 and STAT3 offer good options for using C/EBPβ as a therapeutic target. We will be exploring the aforementioned avenues in our future studies on C/EBPβ-targeted therapy.

In summary, C/EBPβ is an important regulator of the HR pathway and promotes PARPi resistance in ovarian cancer. Our research provides evidence that C/EBPβ could be exploited as an indicator of PARPi responsiveness and a therapeutic target. C/EBPβ detection holds great potential in predicting and tracking PARPi responsiveness during treatment, which warrants further investigations. Targeting C/EBPβ could induce HRD and rescue PARPi resistance, which shows promise for extending the benefits of PARPi to a wider population.

## Materials and methods

### Clinical samples and primary culture

All clinical samples were obtained with signed informed consent from the Gynecology Department at Tongji Hospital (Tongji Medical College, Huazhong University of Science and Technology). The study was supervised by the Ethical Committee of Tongji Medical College (reference number: S1251). All the patients included were diagnosed with HG-SOC and had no prior history of chemotherapy or radiotherapy. Six tissue samples (Patient 1# − 6#) obtained after fresh frozen pathological examination were used for primary culture as reported previously [50]. The epithelial content reached 80% after ten times of serial subculture [50], and was confirmed using EPCAM staining. Six paired samples were obtained at initial surgery and subsequent biopsy. These six patients had HR-proficient HG-SOC and had undergone several rounds of olaparib treatment until disease progression. Patients characteristics were summarized in Supplementary Tables 6 and 7.

### RNA-sequencing and online database analysis

RNA-sequencing was performed by BerryGenomics (China). Each sample had eight biological replicates unless otherwise indicated. DEGs were identified as reported by Schurch et al. using the edgeR (exact) algorithm [51]. Detailed information of RNA-sequencing was described in supplementary information.

Cistrome Data Browser (DB) and JASPAR were used to predict the binding affinity and profile of C/EBPβ [52, 53]. The correlation between C/EBPβ and DDR genes and their gene expression profiles in TCGA dataset were assessed using GEPIA [54], which is an online database containing RNA-sequencing expression data of 9 736 tumor samples and 8 587 normal samples. The relationship between C/EBPβ and olaparib was explored in DepMap (https://depmap.org/portal/), which provides an overview of dependencies operative in cancer cells for new and effective targeted therapies development.

### Primers and plasmid constructs

Primers used in Real-time quantitative reverse transcription PCR were listed in Supplementary Table 8. Primers used in ChIP-qPCR were listed in Supplementary Table 9. Detailed information of the promoter regions used in luciferase reporter assay was depicted in Supplementary Table 10.

### Animal studies

Female NOD-SCID mice (age, four weeks) were purchased from Beijing HFK Bio-Technology Co. Ltd (China) and housed at an accredited facility at Tongji Hospital. The animal experiments were approved by the Committee on Ethics of Animal Experiments of Tongji Hospital. Stably transfected cells (1 × 10^6^) were subcutaneously injected into mouse flanks in a mixture of phosphate-buffered saline (PBS) and Matrigel (Corning, USA) [27]. When the tumor size reached 200 mm^3^, the drug administration schedule reported by Yasukawa et al. was used [55]. The mice were randomly assigned to two groups (six mice per group): (1) olaparib (30 mg/kg per body weight, dissolved in 200 µl sterilized PBS); (2) vehicle (200 µl sterilized PBS containing an equal concentration of dimethyl sulfoxide [DMSO]). The chemicals were intraperitoneally administered every 2 days for 40 days. The tumors were inspected in living mice by optical imaging of luciferase activity with the IVIS SPECTRUM system (Caliper, Xenogen, USA). The total flux was quantified. After imaging, mice were sacrificed for tissue harvest. Survival analysis was also performed, with survival cut-off criteria defined as tumor volume reaching 1 500 mm^3^ or an observation duration of 90 days.

### Statistical analysis

A two-sided Student’s *t* test was used to compare differences between groups unless otherwise indicated. Correlation analyses were performed using Pearson’s correlation test. For the Venn diagram, the chi-squared test was used to evaluate the statistical significance. By Kaplan–Meier plot of survival, Log-rank test was adopted to examine the difference. All experiments were repeated three times unless otherwise indicated. Data were analyzed and plotted using GraphPad Prism 5 (GraphPad Software, San Diego, CA) and presented as mean ± SEM. Significance was assessed at the level of *P* < 0.05.

## Supplementary information

Supplementary Information

Supplementary Table 1

Supplementary Table 4

## Data Availability

The RNA-sequencing dataset was deposited to GEO with accession number GSE153867.
