# Supplementary material for: C/EBPβ promotes poly(ADP-ribose) polymerase inhibitor resistance by enhancing homologous recombination repair in high-grade serous ovarian cancer
Source: Oncogene. 2021 May 8;40(22):3845–58. doi: 10.1038/s41388-021-01788-4 (PMC8175237; doi:10.1038/s41388-021-01788-4)
Supplement: Supplementary file 1 — Supplementary Information [file 41388_2021_1788_MOESM1_ESM.pdf]

Supplementary Information

C/EBPβ promotes poly(ADP-ribose) polymerase inhibitor resistance by enhancing homologous recombination repair in high-grade serous ovarian cancer

Jiahong Tan[1,2, †], Xu Zheng[1,2, †], Mengchen Li[1], Fei Ye[3], Chunyan Song[1,2], Cheng Xu[1,2], Xiaoxue Zhang[1,2], Wenqian Li[1,2], Ya Wang[1,2], Shaoqing Zeng[1,2], Huayi Li[1,2], Gang Chen[1,2], Xiaoyuan Huang[1,2], Ding Ma[1,2], Dan Liu[1,2, *] and Qinglei Gao[1,2, *]

[1]Cancer Biology Research Center (Key Laboratory of the Ministry of Education), Tongji Hospital, Tongji Medical College, Huazhong University of Science and Technology, Wuhan 430030, People's Republic of China.

[2]Department of Gynecology and Obstetrics, Tongji Hospital, Tongji Medical College, Huazhong University of Science and Technology, Wuhan 430030, People's Republic of China.

[3]Department of Neurosurgery, Tongji Hospital, Tongji Medical College, Huazhong University of Science and Technology, Wuhan 430030, People's Republic of China.

Content:

Supplementary methods and materials

Supplementary Table 1-10

Supplementary Figure s1-s17

Supplementary methods and materials

Cell culture and transfection

The OV2008 and C13* cell lines were gifts from Professor Benjamin K. Tsang of the Ottawa Health Research Institute (Canada) [1]. A2780, SKOV3, NIHOVCAR-3, OVCAR-4, OVCAR-8, OV90, CAOV3 and MDA-MB-436 were purchased from ATCC (USA) [1]. MDA-MB-231 were obtained from the Chinese Academy of Science cell bank (China). Cells were maintained in culture media (OV2008, C13*, A2780, NIHOVCAR3, OVCAR-4, and OVCAR-8 in RPMI-1640 [Gibco, USA]; SKOV3 in McCoy's 5A [Gibco]; OV90 in MCDB 105 and M199 [Sigma-Aldrich, USA]; CAOV3 and MDA-MB-231 in Dulbecco's Modified Eagle Medium [Gibco]; MDA-MB-436 in Leibovitz's L-15 medium [Gibco]) supplemented with 10% fetal bovine serum (Gibco) in a humidified atmosphere of 5% CO_2, routinely checked for mycoplasma contamination (Plasmo Test™-Mycoplasma Detection Kit, InvivoGen, USA), and used in less than 20 passages. sv40-Puromycin-CMV-CEBPB and shRNA lentiviruses (GeneChem, China) were used to manipulate C/EBPβ expression. siRNAs targeting CEBPB, BRCA1, BRIP1, BRIT1, and RAD51 were purchased from RiboBio (China). Lipofectamine™ 3000 Transfection Reagent (Invitrogen, USA) was used according to the manufacturer's guidelines.

Reagents and antibodies

Olaparib, AZD2461, BMN673, and Q-VD-OPh were purchased from MedChemExpress (China). Anti-C/EBPβ (ab18336, Abcam, UK) and mouse IgG (ab18413, Abcam) were used for ChIP. The primary antibodies used were as follows: anti-C/EBPβ (ab53138, Abcam), anti-BRCA1 (22362-1-AP, Proteintech, China), anti-BRIP1 (ab151509, Abcam), anti-BRIT1 (11962-1-1AP, Proteintech), anti-RAD51 (ab133534, Abcam), anti-γH2AX (ab26350, Abcam),

anti-GAPDH (60004-1-Ig, Proteintech), anti-Caspase3/p17/p19 (19677-1-AP, Proteintech), anti-Ki-67 (27309-1-AP, Proteintech), anti-β-actin (60008-1-Ig, Proteintech), anti-EPCAM (21050-1-AP, Proteintech), anti-Caspase8 (A0215, Abclonal, China), anti-PARP1 (A19596, Abclonal), anti-GSTP1 (15902-1-AP, Proteintech), anti-NQO1 (11451-1-AP, Proteintech), and anti-PAR (4335-MC-100, Trevigen, USA).

Immunohistochemistry

Formalin-fixed paraffin-embedded tissue sections were analyzed using an Avidin-Biotin Complex (ABC) Vectastain Kit (Zsgb-Bio, China) as per the manufacturer's instructions. For quantification of protein expression, an immunoreactivity-scoring system (HSCORE) was used [1]. All slides were scored by two investigators, who were blinded to all clinicopathological variables.

Western blotting

Western blotting was carried out as reported previously [2]. The signals were detected by enhanced chemiluminescence (ECL, Bio-Rad Laboratories, USA) on a ChemiDoc XRS+ machine (Bio-Rad Laboratories). All experiments were repeated three times.

Real-time quantitative reverse transcription PCR (RT-qPCR)

Total RNA was extracted from cells and tissues using TRIzol reagent (Invitrogen) or RNAiso plus (Takara, Japan) as per the manufacturers' guidelines [3]. RT-qPCR was performed, and mRNA expression was quantified as described previously [1]. Primers used in RT-qPCR were listed in Supplementary Table 8. Each sample had triplicates and all experiments were repeated three times.

Cell viability assay

Cell viability was assessed as previously reported [4]. Briefly, 5×10^3 cells per well were seeded in 96-well plates and treated with serial dilutions of PARPi for 48 hours. Cell viability was determined using Cell Counting Kit-8 (CCK8, Boster, China) according to the manufacturer's guidelines. Each sample had six replicates and all experiments had triplicates.

Generation of an olaparib-resistant cell line

A2780 was cultured under gradually increasing olaparib exposure for nearly four months until the IC50 of the resistant clones was ≥ 30 times that of the parent line [5]. Termination of olaparib treatment and cryopreservation of the cells had very little effect on olaparib resistance.

Colony-formation assay

Colony-formation assay was performed as previously described [6]. After seeding for 24 hours, cells were treated with 3 μM DMSO/olaparib for ten days. For MDA-MB-436, 0.5 μM DMSO/olaparib were used. Colonies of ≥ 40 cells were counted, and colony-formation rates were expressed as the percentage of colonies in PARPi-treated cultures compared with that in control cultures. Each sample had triplicates and every assay was repeated three times.

Immunofluorescence staining

Immunofluorescence staining was performed as previously reported [5]. Cells were treated with 5 μM DMSO/olaparib for two days and then used for experiments. Images were captured using a fluorescence microscope (Olympus, Japan). Results were obtained from three independent experiments.

Flow cytometric analysis

Apoptosis was analyzed using BD Pharmingen™ FITC Annexin V Apoptosis Detection Kit I (BD Biosciences, USA) according to the manufacturer's instructions on the BD FACS Calibur

system (BD Biosciences). A2780 and A2780 OlaR cells were treated with 5 μM DMSO/olaparib for 48 hours and then used for experiments. C13* shCON/shCEBPB cells and OV2008 CON/CEBPB cells were treated with 20 μM Q-VD-OPh for 48 hour and then used for experimental analyses. The cell cycle was analyzed as previously reported [7]. Data were analyzed with CellQuest software as described previously [1]. Each sample had triplicates and all experiments were repeated three times.

ChIP-qPCR

ChIP was performed using the ChIP-IT High Sensitivity® Kit (53040; Active Motif, USA) according to manufacturer's guidelines. DNA purified using the QIAquick PCR Purification Kit (Qiagen, Germany) was quantified using RT-qPCR. Primers used in ChIP-qPCR were listed in Supplementary Table 9. Each sample had triplicates in immunoprecipitation and the experiments were repeated two times.

Luciferase reporter assay

The promoter luciferase reporter plasmids were purchased from TSINGKE Biological Technology (China). Detailed information of the promoter regions used in luciferase reporter assay was depicted in Supplementary Table 10. Renilla luciferase control reporter vector pRL-TK was used as a control. After seeding in 24-well plates, cells were co-transfected with the indicated reporter plasmid and pRL-TK by Lipofectamine™ 3000 (Invitrogen, USA) for 24 hours, then harvested for luciferase activity assays using the Dual-Luciferase® Reporter Assay System (Promega, Germany). Each sample had triplicates and the assay was repeated three times.

HR reporter assay

Cells were plated in 6-well plates and co-transfected with pCBAScel plasmid (#26477, Addgene, USA) and pDRGFP plasmid (#26475, Addgene) by Lipofectamine™ 3000. After a 72 hours' incubation, cells were collected and GFP expression was detected by flow cytometry (BD FACS Calibur). Each sample had triplicates and all experiments were repeated three times.

Measurement of ROS

ROS levels were detected using the Reactive Oxygen Species Assay kit according to manufacturer's protocols (Yeasen Biotech Co. Ltd, China). Briefly, cells were harvested and incubated with 10 μM 2,7-dichlorodi-hydrofluorescein diacetate (DCFH-DA) at 37 °C for 30 minutes. ROS distribution was measured by flow cytometry (BD FACS Calibur). Each sample had triplicates and every assay was repeated three times.

Alkaline comet assays

Cells were treated with 5 μM DMSO/olaparib for two days and then DNA damage was analyzed. Comet assays were performed using the CometAssay Kit (4250-050-K; Trevigen, USA) as previously reported [1]. DNA damage was expressed as 'DNA in tails (%)'. The average percentage of the DNA in tails was measured for ≥ 30 cells per sample using CometScore1.5 Software (Tritek). Results were obtained from three independent experiments.

RNA-sequencing

RNA was qualified using Agilent 2100 and quantified using Qubit. A total of 2 μg RNA per sample was used for library construction (VAHTS mRNA-seq v2 Library Prep Kit, Illumina). The final library size was about 350 bp per sample. After 150-bp paired-end sequencing on the Illumina Novaseq6000 platform, ≥ 6 Gb data per sample were obtained. Clearly filtered reads were mapped to hg38 using TopHat. HTseq was used to count the reads numbers mapped to

each gene. All diagrams including heatmap, Venn diagram, and pie chart were drawn using R. GO and KEGG enrichment analysis were implemented using the GOseq R and KOBAS 3.0 package.

References

1 Liu D, Zhang XX, Li MC, Cao CH, Wan DY, Xi BX *et al*. C/EBPbeta enhances platinum resistance of ovarian cancer cells by reprogramming H3K79 methylation. *Nat Commun* 2018; 9: 1739.

2 Wei X, Liu Y, Gong C, Ji T, Zhou X, Zhang T *et al*. Targeting Leptin as a Therapeutic Strategy against Ovarian Cancer Peritoneal Metastasis. *Anti-cancer agents in medicinal chemistry* 2017; 17: 1093-1101.

3 Fu F, Wang T, Wu Z, Feng Y, Wang W, Zhou S *et al*. HMGA1 exacerbates tumor growth through regulating the cell cycle and accelerates migration/invasion via targeting miR-221/222 in cervical cancer. *Cell Death Dis* 2018; 9: 594.

4 Kim H, George E, Ragland R, Rafail S, Zhang R, Krepler C *et al*. Targeting the ATR/CHK1 Axis with PARP Inhibition Results in Tumor Regression in BRCA-Mutant Ovarian Cancer Models. *Clinical cancer research : an official journal of the American Association for Cancer Research* 2017; 23: 3097-3108.

5 Sun C, Yin J, Fang Y, Chen J, Jeong KJ, Chen X *et al*. BRD4 Inhibition Is Synthetic Lethal with PARP Inhibitors through the Induction of Homologous Recombination Deficiency. *Cancer Cell* 2018; 33: 401-416 e408.

6 Zhang X, Liu D, Li M, Cao C, Wan D, Xi B *et al*. Prognostic and therapeutic value of disruptor of telomeric silencing-1-like (DOT1L) expression in patients with ovarian cancer. *J Hematol Oncol* 2017; 10: 29.

7 Zhang Z, Li X, Han Y, Ji T, Huang X, Gao Q *et al*. RAD54B potentiates tumor growth and predicts poor prognosis of patients with luminal A breast cancer. *Biomed Pharmacother* 2019;

118: 109341.

Supplementary Table 1. Differentially expressed genes after C/EBPβ knockdown.

See attached Excel file for detailed information.

Supplementary Table 2. The effects of C/EBPβ knockdown on DDR-related KEGG pathways.

**Term**	**Database**	**ID**	***P* value**
Homologous recombination	KEGG PATHWAY	hsa03440	0.03423
Base excision repair	KEGG PATHWAY	hsa03410	0.41685
Nucleotide excision repair	KEGG PATHWAY	hsa03420	0.63844
Mismatch repair	KEGG PATHWAY	hsa03430	0.80044
DNA replication	KEGG PATHWAY	hsa03030	0.71632
Fanconi anemia pathway	KEGG PATHWAY	hsa03460	0.09940

Supplementary Table 3. Enriched DDR-related Gene Ontologies after C/EBPβ knockdown.

Term	Database	ID	*P* value
Cellular response to DNA damage stimulus	Gene Ontology	GO:0006974	1.54E-07
DNA damage response, signal transduction by p53 class mediator resulting in cell cycle arrest	Gene Ontology	GO:0006977	3.49E-05
Intrinsic apoptotic signaling pathway in response to DNA damage by p53 class mediator	Gene Ontology	GO:0042771	0.00029473
Intrinsic apoptotic signaling pathway in response to DNA damage	Gene Ontology	GO:0008630	0.0012286
Intra-S DNA damage checkpoint	Gene Ontology	GO:0031573	0.00268911
Mitochondrial DNA repair	Gene Ontology	GO:0043504	0.00372132
DNA repair	Gene Ontology	GO:0006281	0.00725184
DNA damage response, signal transduction by p53 class mediator	Gene Ontology	GO:0030330	0.01690195
Positive regulation of DNA damage response, signal transduction by p53 class mediator	Gene Ontology	GO:0043517	0.02292315

DNA ligation involved in DNA repair	Gene Ontology	GO:0051103	0.02917673
Mitotic G2 DNA damage checkpoint	Gene Ontology	GO:0007095	0.03342049
Negative regulation of intrinsic apoptotic signaling pathway in response to DNA damage	Gene Ontology	GO:1902230	0.04125086

Abbreviation: GO, Gene Ontology.

Supplementary Table 4. Differentially expressed genes between A2780 and A2780 OlaR.

See attached Excel file for detailed information.

Supplementary Table 5. Prediction of the binding profiles of C/EBPβ in JASPAR.

Motif	Sequence	Predicted sequence	Score	Relative score	Start	End	Strand
MA0466.1	BRCA1	gctttcaaaat	8.63559	0.910459	1471	1481	+
	BRIP1	aattacttcat	7.40976	0.897609	812	822	+
	BRIT1	gatttcatcac	13.076	0.957004	171	181	+
	RAD51	ggtttcaccac	11.4691	0.940161	1111	1121	+
MA0466.2	BRCA1	gtgatgcaat	8.39749	0.898549	1821	1830	+
	BRIP1	tttatgcaat	8.8804	0.905088	2512	2521	+
	BRIT1	attacaccac	7.837	0.89096	485	494	+
	RAD51	cttacgcaaa	8.73782	0.903157	1879	1888	+

Supplementary Table 6. **Characteristics of patients used in olaparib sensitivity detection.**

Patient No.	Age at diagnosis	FIGO stage	Histology	Ascites	HRD status
1#	55	IIIC	HG-SOC	Yes	positive
2#	61	IIIC	HG-SOC	No	positive
3#	48	IIIC	HG-SOC	Yes	negative
4#	49	IIIC	HG-SOC	Yes	negative
5#	55	IIB	HG-SOC	Yes	negative
6#	52	IIIC	HG-SOC	Yes	negative

Abbreviation: FIGO, International Federation of Gynecology and Obstetrics; HG-SOC, High-grade serous ovarian cancer.

Supplementary Table 7. Characteristics of patients treated with olaparib.

Patient No.	Age at diagnosis	Histology	FIGO stage	Ascites	Debulking surgery	Olaparib treatment duration	HRD status
1	43	HG-SOC	IIIC	yes	yes	8 months	negative
2	47	HG-SOC	IV	yes	yes	9 months	negative
3	39	HG-SOC	IIIA	no	yes	18 months	negative
4	42	HG-SOC	IIIC	yes	yes	15 months	negative
5	50	HG-SOC	IV	yes	yes	6 months	negative
6	46	HG-SOC	IV	yes	yes	12 months	negative

Abbreviation: FIGO, International Federation of Gynecology and Obstetrics; HG-SOC, High-grade serous ovarian cancer.

Supplementary Table 8. Primers used in RT-qPCR

Target	Forward	Reverse
BABAM1	CACTCGCTCCAATCCTGAAGG	CTGAAGTGTTGAGGCTCCCAT
BRCA1	GAAACCGTGCCAAAAGACTTC	CCAAGGTTAGAGAGTTGGACAC
BRIP1	CTTACCCGTCACAGCTTGCTA	CACTAAGAGATTGTTGCCATGCT
BRIT1	ATGTAGTGGCCTATGTTGAAGTG	CCACAAGCTGTGTTGTAAATGTC
CDK12	CTAACAGCAGAGAGCGTCACC	AAAGGTTTGATAACTGTGCCCA
CEBPB	TCCAAACCAACCGCACAT	GAGAGGGGCAGAGGGAGAAGCAGAG
DNA2	AGAGCTGTCCTGAGTGAAACT	GAAACACCTCATGGAGAACCG
FANCB	ATGAAGGATGGCCTAAGGGTC	ACACACTAACAACTTTGCCAGT
FANCM	TCGTGACGGTGGTTACAACAC	ACAAGACGAATTGGGCTCTTC
GAPDH	ACAACTTTGGTATCGTGGAAGG	GCCATCACGCCACAGTTTC
POLD4	TATCTATACATTCCCAGCAAGCACCTC	GCCCAGCCATCAGTTTCTTACCA
RAD50	TTTGGTTGGACCCAATGGGG	CAGGAGGGAAATCTCCAGTACAA
RAD51	CAACCCATTTCACGGTTAGAGC	TTCTTTGGCGCATAGGCAACA
RBL1	ATGAAGAACCACCAAAGTTACCA	GCAGCATAGAAGTAAATGATAAGAG
REV1	GATGGAGGAAGCGAGCTGAAA	CCTTCTGCATAGCAGCATCTG
RFC1	TTGAACGAGATGAGGCCAAGT	ACTATCACGACCCATGACAAGAT
USP1	ATGCCTGGTGTCATACCTAGT	CAGTCCCACAAATGGTAACAAGT
WRN	CACAGCAGCGGAAATGTCCT	GAGCAATCACTAGCATCGTAACT
XRCC2	AAGAACCGAATACAGCAGGACAG	TCAAGGGACTCAGCTAGGCAAGG

Supplementary Table 9. Primers used for ChIP-qPCR.

Target	Forward	Reverse
BRCA1-1	GCAGGCACTTTATGGCAAACTCA	CAATCAGAGGATGGGAGGGACAG
BRCA1-2	GCAGGCACTTTATGGCAAACTCA	GGTACAATCAGAGGATGGGAGGG
BRCA1-3	TCTAAGGAACACTGTGGCGAAGAC	TCGTAAGAAGAGGTCCCAATCCC
BRIP1-1	AGATTGTAGCCCGATGTCACAGA	CGTAGACCAGCGGAAAGGAAGTA
BRIP1-2	ATTGTAGCCCGATGTCACAGAGC	CGTAGACCAGCGGAAAGGAAGTA
BRIP1-3	AGATAGATTGTAGCCCGATGTCA	TAAATTACCCAGCTTTGCAGTAG
BRIT1-1	CGGCAGTTGAGTTTCTATGCTAAT	AGAGTCACCTAAACGTCCGATGC
BRIT1-2	TACAGGCAGAAACTAAGGCATCG	CTCACGGTCAAACTCTACAAGACAC
BRIT1-3	AGCGGCAGTTGAGTTTCTATGCT	AGAGTCACCTAAACGTCCGATGC
RAD51-1	CCGTGGTTAGCCTCGAACTCCTA	TTTACAGACTGCCCTCTTCCCTTT
RAD51-2	AGATACTGCCGAAACAAACCACAA	CCACGACTCCCAAGAGGTAATGC
RAD51-3	ATACTGCCGAAACAAACCACAAGA	CCACGACTCCCAAGAGGTAATGC

Supplementary Table 10. Promoter regions used in plasmid construct.

Gene	Sequence
BRCA1	ctgattggtgcatttgcaaaccttgagctagacacagagcactgattggtgcatttacaatcctttagcta<br>gacacagaagttctccaagtgcccaccagattagctagatacagagtgctgattggtgcatccccaaa<br>ccccaagctagacacagagtgctgactggtgcatataaaatcctcaggctagacataaaagttttccaa<br>gtccccatctgactcaggagcccagctggcttcacctagtggatcctgcgcagggctgtgccgggcg<br>cctgcactcctctcagcccttgggcagtcgatgggaccgggcgctgaggagcagggggcggtgcc<br>cgtcggggaggctcaggccacgctggagctcacaggggttgggagggggctcgggcatggcggg<br>ctgcaggtcctgagccttgccctgtgcagggcggctggggcccggtgagaattcaagcggggtgca<br>ggcgggccggcagtgctgggggacccggcgcaccctctgcagctgctggcccgggtgctaggcc<br>cctgactgcccggggccgggggtgcggggcccgctgagcccgcgcccacctggaactcgcgctg<br>gctggcgagcgctgcgcgcagccccagttcccacacccgcctctccctccacacttccccgcaagc<br>agagggagccggctctggcttcggccagcccagagaggggcccccacagcgcagtggcgggctg<br>aagggctcctccagcacggccagaatggacgccaaggccgaggaggcgccgagagcgagcgag<br>ggctgctagcacgttgtcacctcgcattctgaaccacagactctccaactctccggcgcttttcgccca<br>ctcggtccctcagaacacgaagggctctctcatcctgtcactaaaacgattagctgtccggagacacg<br>gaaaaagtcgcccctcttctttgcaggattcctcccttgaacttctccaaaccctcttagtgtgacgtgac<br>cccacccctagctaacccaggctgcttccttaccagcttcccgccccctggggaggcggcaatgcaa<br>agaccgtccgctgccagctctgccgctatctctgtggggtgaatctaacatggcggacaaagacagta<br>actagtcccgtttctccgcgttttcgccaagaagattggctcttaccacttgtccctcaaaacgaccacc<br>ccattgactggtggcgattgcgtcgacggagacggggcaaaagcaagctgaacccgaaaaataaca<br>aacactggggctgaggggtggaactacgagtgcgcagacatgggccagagcgcatttcccctgccc

	caggcaaattcggcgctcactgcgtccccgcaggccactgaccttacaagactacttgccccagact<br>cctggggctggatgggaattgtagtctccctaaagagttgtacgtatctttttaaggcctagtttctgcttt<br>caaaatacgaaaacataacactccagtccataactgttgacaagtacaagcgcgcacaggtctccaat<br>ctatccactggatttccgtgagaattgtgcccgctctggtattggatgttcctctccataagactacagttt<br>ctaaggaacactgtggcgaagacctttcattccgcaacgcatgctggaaataattatttccctccacccc<br>cccaacaatccttattacttatatttaccgaaactggagacctccattagggcggaaagagtgggggat<br>tgggacctcttcttacgactgctttggacaataggtagcgattctgaccttcgtacagcaattactgtgat<br>gcaataagccgcaactggaagagtagaggctagagggcaggcactttatggcaaactcaggtagaa<br>ttcttcctcttccgtctctttccttttacgtcatccgggggcagactgggtggccaatccagagccccga<br>gagacgcttggctctttctgtccctcccatcctctgattgtaccttgatttcgtattctgagaggctgctgct<br>tagcggtagccccttggtttccgtggcaacggaaaagcgcgggaattacagataaattaaaactgcga<br>ctgcgcggcgtgagctcgctgagacttcctggacgggggacaggctgtggggtttctcagataactg<br>ggcccctgcgctcaggaggccttcaccctct
BRIP1	aaatgtgtaaatatattagtaaatgttaatattcatgtattttaagttaaggttataaaatttgtcacaatgtgtt<br>tttttattcaagtgaaaacagatgtgtgcagctattttgaatattggtttataaacattcatattctttatcaaa<br>cgaacctgtagtttttgtgtttcattccactagagttaacctgaagggaacggttcaagggactgtattcg<br>aggtctgtatggctccagggcctacgtgtatccctttctgttatgccacatacattaaatggcaatgcaag<br>gtgagagccagttgaaactcttagtaatttaggagaggaaaatgagagtagttatactagagcagttac<br>tcaaaagagttgattttttttttttttgagacggagtctcgctctgtctcccaggctggagtgcagtggcgc<br>catctcggctcactgcaagctccgcctcctgggttcacgccattctcctgcctcagtctcccgagtagct<br>gggactacaggcgcccgccaccacgcccggctaatttttgtatttttagtagagacggggtttcaccg<br>tgttagccaggatggtcttggtctcctgacctggtgatccgcctgcctcggcctcccaaagtgctggga

ttacaggcgtgagccactgcgcccggccaagagttgatgttttaaaaattataaaagagaaatgacaat

ttgaaggtatttttaaggatttatttttaaacattatccttcctttcattgtcttaacagtaaattacttcatctttg

ctcattcatatgtattttcacattactgaatccctaacacccagtttggggtttgctttgcctttttcacttaac

cttatatgggatttttcttgcaagtttaatttttataagtttattggcttcataatgtattgaattcatctgtcata

accacctccatattactagactgggaagttcataattttttcagtattaccaatagatattattgtcaattgc

ggtatcacattttctttcgtttagattacgtctttaggataaatatctggagctcagattactaggtcaaagg

gtagagtttatttttatgactacaagttttgtcatttttggtaagatatatcacaattgacaatgaggcaaaca

aatcaactagccccctcctgctctttcttcccagcaaactccttccccaaactagttacttgaatttgagaa

acagtatgaggttttgccttttggagacattattatttgagccaagtttaggttatacagatttttttaaaaaaa

taacatgatttggcaagatcatgatgaacctccttttctttgagtaaattaaagtactactggcaaataata

aactggtcttaggagggatggaaggcggataagccttaatggaagtagaagtgatctgatactggag

accctgagagcttgtctctggcttattttagcatccgggaaagccttgaatattatgaaagatagattgta

gcccgatgtcacagagccttcttacttaagtttggctcagcagtctggcaattcccgcctgagtcttcgct

atcctggctactgcaaagctgggtaatttaaattgagtggcactgataaatgctcaaagaacttggcaa

agcttaaagatacttcctttccgctggtctacggacgtgaagaatgagacttccagtttccaaggaatttg

ctgcatagactctatcgccggtttcaaaagtcaaactgagacgactttctactttaaacaaacactaggg

atttgctggagtggcgacaaccttccgccgcgtaccttgagcccttgttttttttttttttttcggagacttc

cattggatgccgaagttctcgccccctcacctcgcaccgggactggttgattcctggccttggcgggt

gattggtcgagctcgctgggctattgggcgctgggagtcgagggggcgggaggcgggaattcgtct

cgggttgtgtggttgaggggtctggtgggtcgaggaaaggtaacggcggccccagtcctgcacaca

aggccggggaagtagcagcacccccaggaagagggaggaggaagggctcgtgccctttcttctctt

ccagggctccgctttatttgctctcagaagtcggtttcctttccttttcttcagtgaat

BRIT1	caatctcaatcaaaatcccagcagtattttttgtgcaaaatgagaagtcgactctaagatttaaaaggaa<br>atctgaagaatctagaagatacaaaataaccttgaaaaataaagttgtaggacataaactatctgatttca<br>tcacttatttatatagctacaataatcaaaacagcatggtgctggcagcaaaaagacaaatagctcaatg<br>gaacacaataggaagcctaaaatgaaacacatacatatgcaacacagattttgatgtaagcacaaagg<br>aaatgcagtagagacaaaaataactttttaataaatgatgctggaacatttggatatgtatacatgcaaaa<br>aaatgaactttggtccctatcccataccgtatacaaaaattaattaaaagcagatcttatcctttgagtcca<br>gtaggttgaggctgcagtgagctgtgattacaccactgcattccagcctgggcaacggagtgagaaa<br>ctgcctggagaaaaaaaaaaaaaaaaaagtagaacctagacctgatatacaacctaaagcagtaatattt<br>ctagaagaaatcctaggagaaaatatttgtgatcgtggagatgaagaatctatcaaatactaaactttttt<br>accaccttgaccaaaagtaattggtttatatacttcatcatatcatttaattcaaaatctacagagatcaatg<br>tcactttctcagtaaaagtacgtgagtcttcaatgatgccctgaactcacactcccaagtaaaccataac<br>accatatttccagagtagagtttattagaacaataactggtgataatgataaatattgatcaaagactgag<br>cctaggaagtgggttttttgaggctgcatatactcaaggcaattcttcagaaccacagagggctcattg<br>gatcctattaaaagctgagagtgaatgaataaacagataaaacagagacctgagtagacggtagtcg<br>atattcttttacatgtattctacctctagattccatagaaagaactaaaagtacatgaatttcactaccaaca<br>tctccatcagttaccagctgtatcaccttggatcagtcaggtaacctcccgcgaatctggttgcttccgg<br>ggcaggggatccgcgggctgcaggtttgagcctggttgccggcagggtggagcagctggagggc<br>caagcctttgagctccagggggggtggccgggacagtgggtagtgccagccgatcggcgtcctgg<br>ggattgcctgaatgtgaggtctgggttcaccccgcggtgacctgagtcctgggatgcccctacaggg<br>atttgctgcctcagggatccgaagtctctttcattcccttactggggatttgaggtctggaggtactcctg<br>cgggggtctgagatctcggggtcaccctgtgggggtctgaagcctcggggtccccgcgtggggtct<br>gaggtatcagagtcccctccgttgggtctgaggtctcggggtcccccatccccgggatcggaggtcc

	ggctccccggagcaggcagggcggtgcgtctggccctgaacagtaacgtggcgcgccagcccca<br>ggtggtgtcgggctagggggggcataacggtgccgaaagtccgcacaaagccgtccgctggggtcc<br>cgccgcgtccgcgagggaatgactgtgccccctccccttcctgatcctcagctcaggtgagcccaga<br>tgaggcgccgggtagcttctaagtcactaatggaaatagaaggctaattcaggggttaggggccgtc<br>gtcctccttactcccaggagaagagaaaaacccacggcccagcagccagaggcgcggcgaggcg<br>gaatcgggccccctccccgggggctcagctccctccagcctcccgcctcacctacagagaaatccc<br>ggaaacgcggattcagcggagcgcggtgacggcggcgcgctcaccccgcgcatgcccagtgccc<br>gcgcgcgccgccaggctcgcaagcaccgcgtaggccagctggccggatcccgccgtctgtcatgg<br>cggcccccatcctgaaaggtgaggtacttcctgctgcctgctccagcagcgggagtttgaggaccgg<br>cacccctcgtcgcgggcgcactcgggggatcccgtgggaggagccccgctcgcccctccctcgct<br>gcctgtctcccccagaccccctg
RAD51	ggagggaaaatagatctaaccaaatgacttgtcttcttaaaaacttcaatgatttcttcagtacctagaga<br>ccaaagctccttaccttttttttttttttgagacggagtcttgctctgttgcccaggctggagtgcagtggc<br>gtgatctcgctcactgcaacctccacctcccgggttcaagcacttctctgcctcagcctcccaaagagc<br>tgggattacaggcatgcaccaccacgcccggctaatttttgtagttttagtagagatggggttttgccatc<br>ttggccaggctggtcttgaactcctgacctcgtgatccgcccaccttggcctcccaaagttctgggatta<br>caggcttgagccaccgcgcctggcctctccttacatgttttgttgttgtcgcttgtcttttgagacaaggtc<br>tcactctgtagcccaggctggagtgcagtggcccatcatagctcactgcagccttgacgtcctgagct<br>caagcagtcctcccacctcagcctccgggatagctgagaccacaggcacaagccaccacgcccag<br>cttattttttttttttttttttttgagatatggggtcccactatgttgcccaggctggtctccaactcctgagctc<br>aagcgatcctcccccaccttggcttcccaaagtgctgggattacaggtgtgagccaccgtgcaggcct<br>tatatgatcttcatatcctgaactaaatgtaaccttccagtttcggcacttgctctggcactttcctccctcg

ccagataatactaatctttaatcatgtagttcgtttccatgcccatactaccctatttgcttataatgtcttcca

cttcgcccaagaatccctactcagctagcttgtggtgttgttttgacacagtctcgctctgtcgcccaggc

tggagtacagcggcgagatctcggttggctgcaacctcctcctgagttcaagcgattctcatgcctcag

cctttcgagtagctaggattacaggcatgtgccacaaaacctggctaatttttgtatttttttactaaagacg

aggtttcaccacgttggccaggtttatctccaacccctgacctcaggtgatccgcctgccttggcctcct

aaactgctgggattactggcgtgaaccaccgcgcccggccctactcagcctttaaaaccggaatcac

gggtcaaaactttctggtaaaccacgatacggtttaggttatgaaattcaatgccccccttcctctgaactc

ctgcaaatctccagtaaagcaccacagattgacgaatattccagccatttccctctcccgtacgctagct

ccatttcccacttctatccatcttctcgagcttcctcagctcctccacctccatgaggcctggaaagcacc

ttgctccaggaatgcgagtaggaggctcagagcgaccagaagtgccaaaagctgacattcagatact

gccgaaacaaaccacaagagcgctagggcccccgctaatagtccagctgcgatggtgagaactcg

cggacccgccggcgatgcatgccgggagatgtagtcccgggccgacgcattacctcttgggagtcg

tggtcttcgatctggtaaacagaagacggcaactcggttaagtcttcccccaccgccccctgaaatccc

tcgccccacccgcgagggactggggtaggagtaggggcgttgccgtggttagcctcgaactcctag

gctcagacgatactctcgcctcggcctcccgagcagctgggactacacgcgtgagccaccgccccc

ggcataaagtttgaattagtccttacgcaaaaagggaagagggcagtctgtaaactcgcgcaggatca

agctctcgagctcccgtcttgggttagcgcgcagggcggaagcggggagaaggcggatccggga

ggcggggatacgttacgtcgacgcgggcgtgaccctgggcgagagggtttggcgggaattctgaaa

gccgctggcggaccgcgcgcagcggccagagaccgagccctaaggagagtgcggcgcttcccg

aggcgtgcagctgggaactgcaactcatctgggttgtgcgcagaaggctggggcaagcgagtaga

gaagtggagcgtaagccaggggcgttgggggccgtgcgggtcgggcgcgtgccacgcccgcg

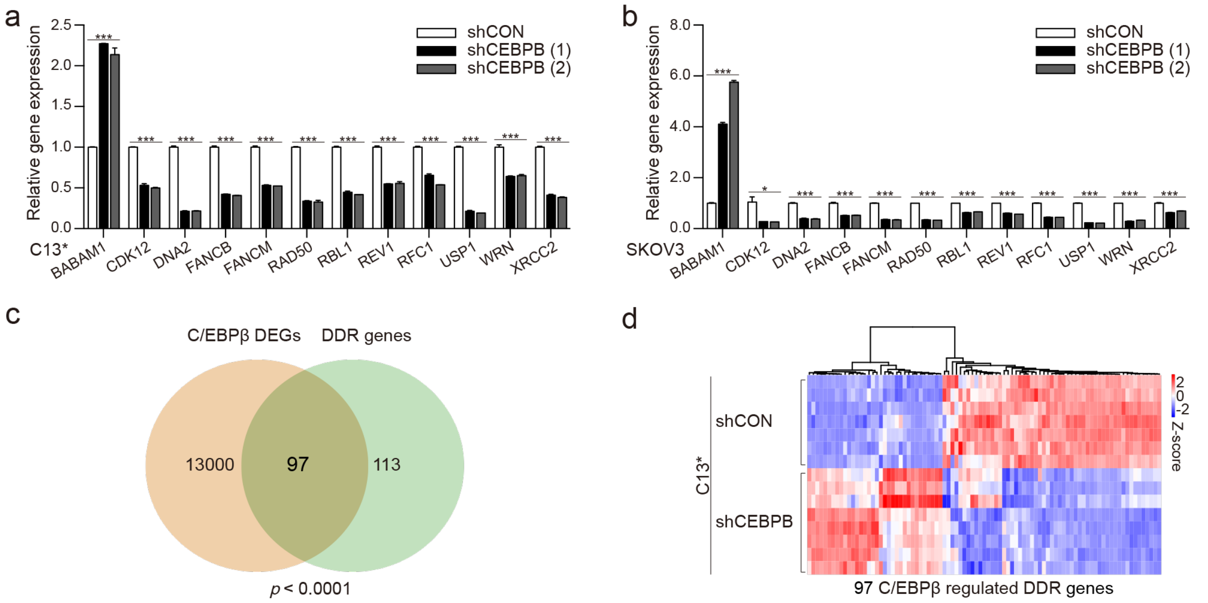

Supplementary Figure s1. Expression variations after C/EBPβ knockdown. RT-qPCR verification of the RNA-sequencing using (a) C13* cells and (b) SKOV3 cells. Each sample had triplicates and data were presented as mean ± SEM of three experiments (Student's *t*-test). (c) Venn diagram of DEGs after C/EBPβ knockdown and DDR genes retrieved from the KEGG database (Chi-squared test). (d) Heatmap of the 97 overlapping genes. *P* value was denoted as * $P < 0.05$, ** $P < 0.01$, and *** $P < 0.001$, "n.s" represents "not significant".

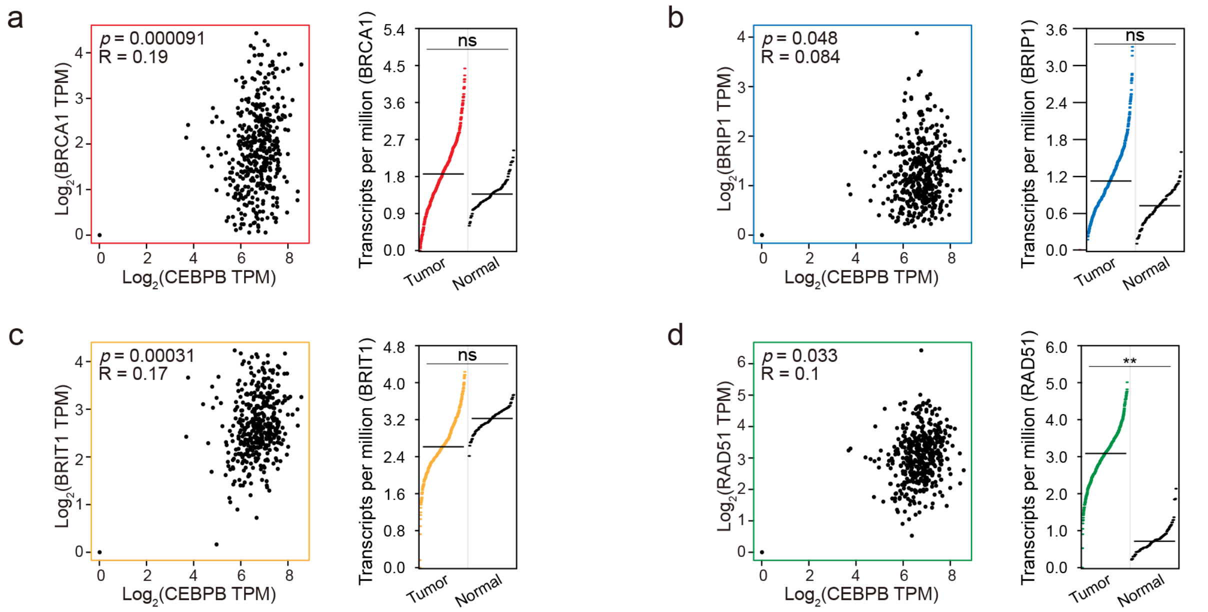

Supplementary Figure s2. Correlation analysis of C/EBPβ and the four representative DDR genes in the TCGA dataset. DEGs after C/EBPβ knockdown overlapped with DDR genes retrieved from the KEGG database. The 97 overlapping genes were further explored in the TCGA dataset using GEPIA. The gene expression of four DDR genes, namely, BRCA1, BRIP1, BRIT1, and RAD51, correlated with C/EBPβ expression, and their expression was depicted (Pearson's correlation test, one-way ANOVA).

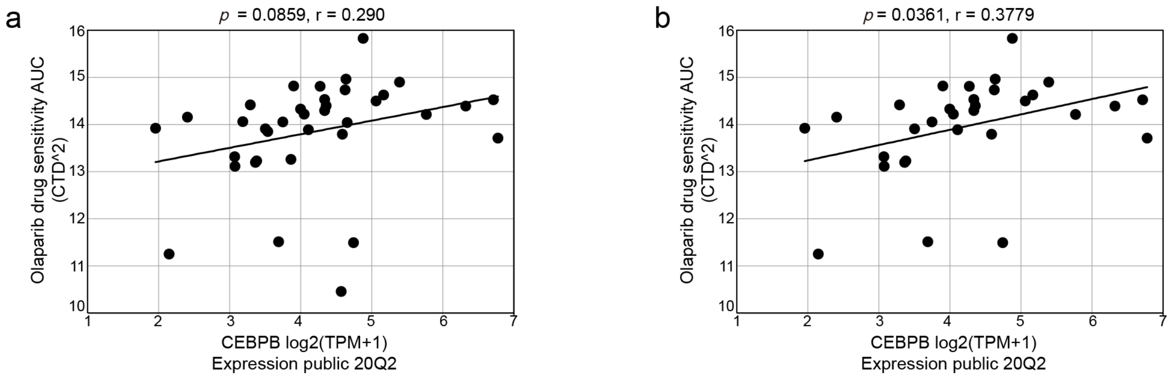

Supplementary Figure s3. Correlation analysis of C/EBPβ and olaparib responsiveness in DepMap. The correlation between C/EBPβ and olaparib responsiveness was assessed in DepMap. Drug sensitivity AUC value was used to evaluate drug resistance. The higher the AUC value, the more resistant the cell line. (a) Correlation analysis of C/EBPβ and olaparib drug sensitivity AUC in all ovarian cancer cell lines included in Expression public 20Q2. (b) The correlation between C/EBPβ and olaparib drug sensitivity AUC was further analyzed in HR-proficient ovarian cancer cell lines. (Pearson's correlation test; AUC, area under the curve)

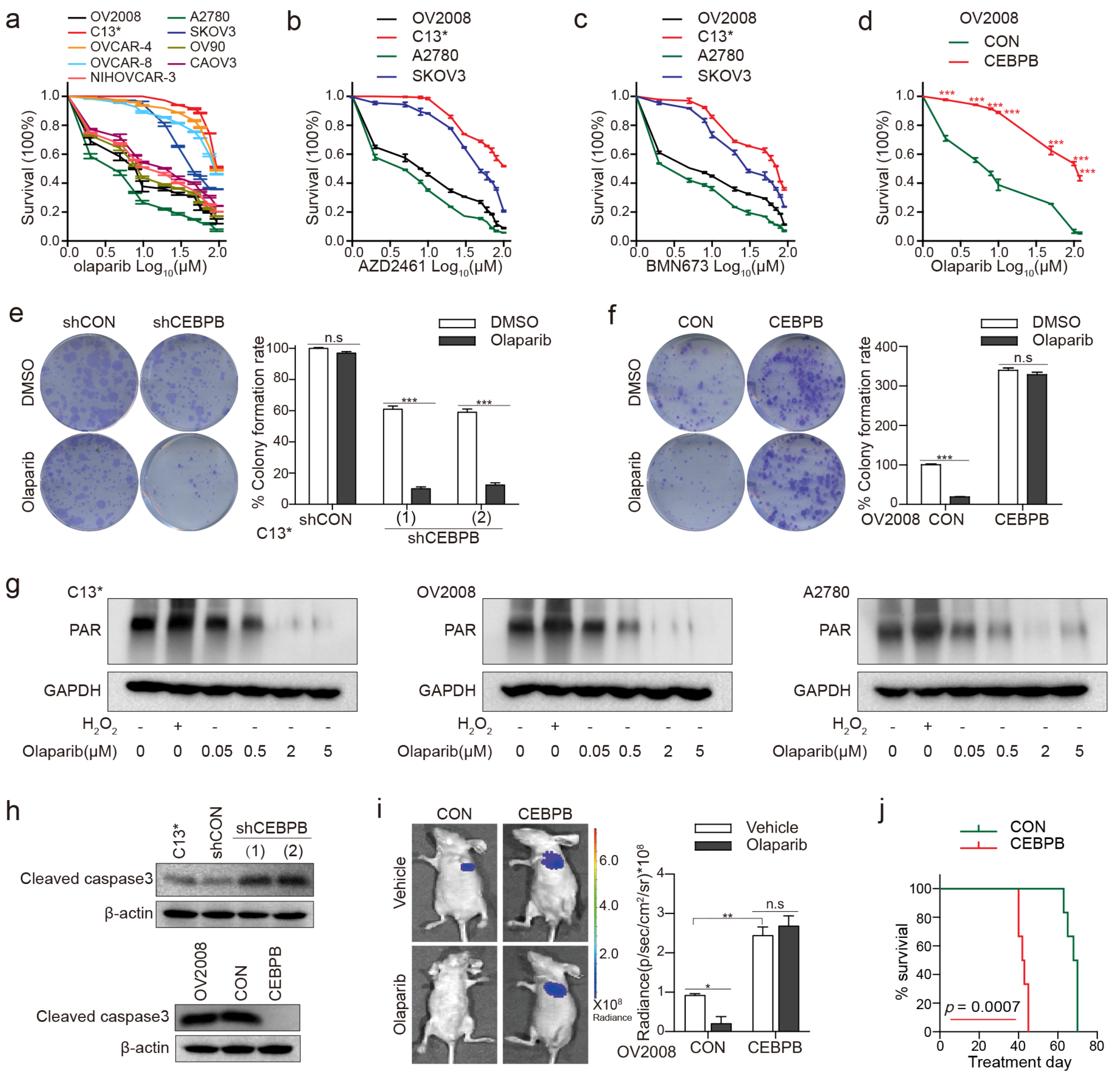

Supplementary Figure s4. C/EBPβ expression is correlated with PARPi responsiveness.

(a) Cell viability assays for cancer cell lines with different olaparib concentrations. Cell viability assays of four cancer cell lines under different concentrations of (b) AZD2461 or (c) BMN673. (d) Cell viability assays for C/EBPβ-overexpressing OV2008 cells (Student's *t*-test). By cell viability assays, all experiments were repeated three times in six replicates. Data were presented as mean ± SEM. (e) Colony-formation rates of C13* after C/EBPβ manipulation and olaparib treatment (Student's *t*-test). (f) Clonogenicity of OV2008 was detected after C/EBPβ overexpression and olaparib treatment (Student's *t*-test). By colony formation assays, the

experiments were conducted in triplicates and error bars indicated mean ± SEM of three assays. (g) Western blotting analysis of PAR in C13*, OV2008, and A2780 under different olaparib concentrations. H_2O_2 was used as the positive control. A representative blot of three experiments was shown. (h) Cleaved caspase3 expression was detected using western blotting analysis after C/EBPβ manipulation in C13* and OV2008 without olaparib exposure. The experiments were repeated three times. OV2008 xenografts models were constructed after C/EBPβ manipulation (six mice per group). (i) Tumors were inspected and tumor burdens were evaluated. Representative images of mouse xenografts were shown (Student's *t*-test). Error bar, mean ± SEM. (j) Survival analysis of mice after tumor inoculation and olaparib treatment (Log-rank test). *P* value was denoted as * $P < 0.05$, ** $P < 0.01$, and *** $P < 0.001$, "n.s" represents "not significant".

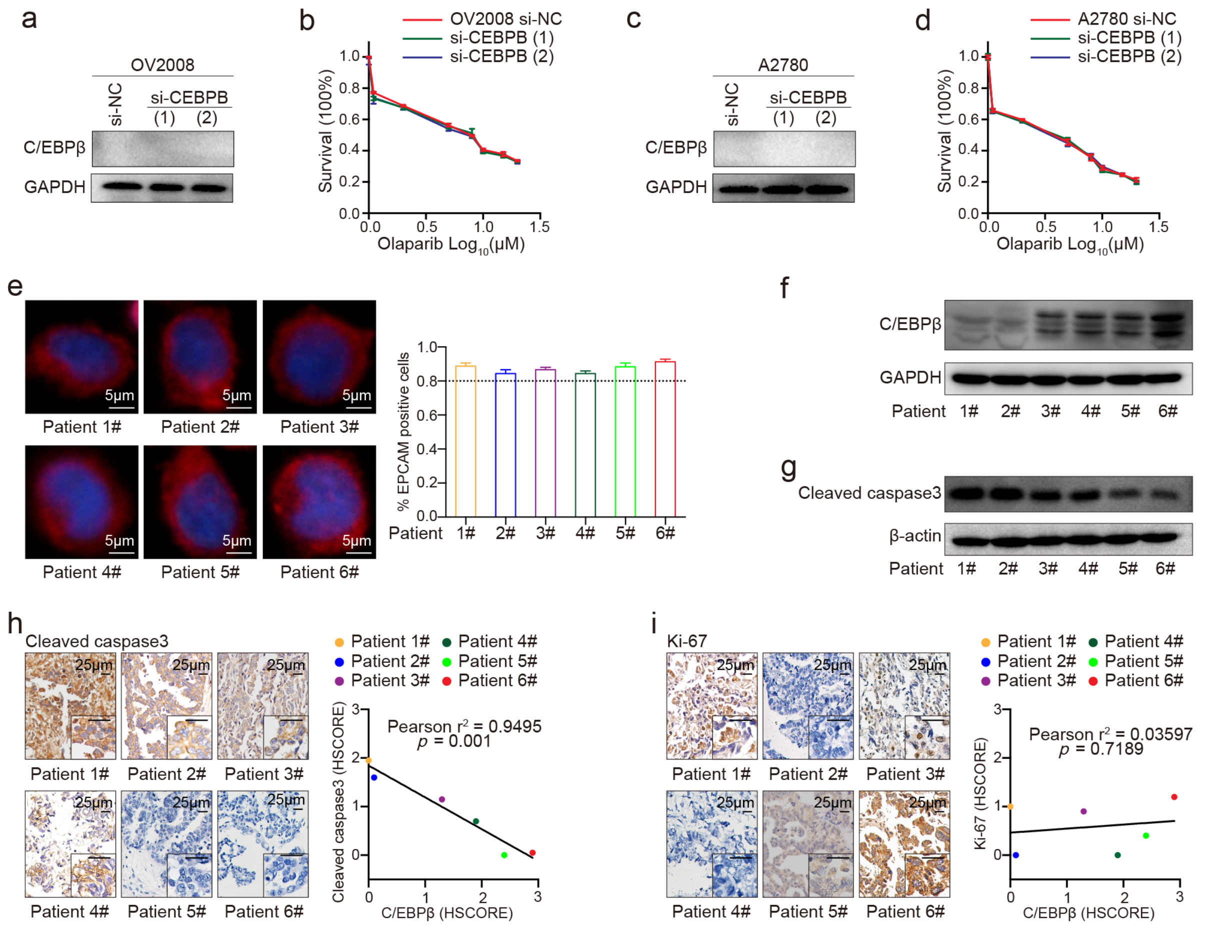

Supplementary Figure s5. Verification of the correlation between C/EBPβ and PARPi responsiveness. (a) siRNA interference of C/EBPβ expression in OV2008. The experiment was repeated thrice and representative images were shown. (b) Cell viability assays of OV2008 after siRNA interference. Each sample had six replicates and all experiments had triplicates. Error bars represented mean ± SEM. (c) C/EBPβ-targeted siRNA interference in A2780. A representative blot of three assays was shown. (d) Cell viability assays of A2780 after siRNA transfection. Each assay was conducted in six wells and repeated three times. Error bars depicted mean ± SEM. (e) EPCAM staining of primary cultures to evaluate epithelial content. Representatives were shown (left), and percentage of EPCAM positive cells was calculated (right). Data were obtained from three independent experiments and denoted as mean ± SEM. Western blotting analysis detected (f) C/EBPβ expression and (g) cleaved caspase3 expression

in primary cultures corresponding to the clinical samples used in Fig. 2h. By western blotting analysis, all experiments were repeated three times. Immunohistochemical analysis of cleaved caspase3 and Ki-67 of the clinical samples used in Fig. 2h was performed. (h) The correlation between C/EBPβ and cleaved caspase3 was analyzed (Pearson's correlation test). (i) Correlation analysis of C/EBPβ and Ki-67 was performed (Pearson's correlation test). *P* value was denoted as * $P < 0.05$, ** $P < 0.01$, and *** $P < 0.001$, "n.s" represents "not significant".

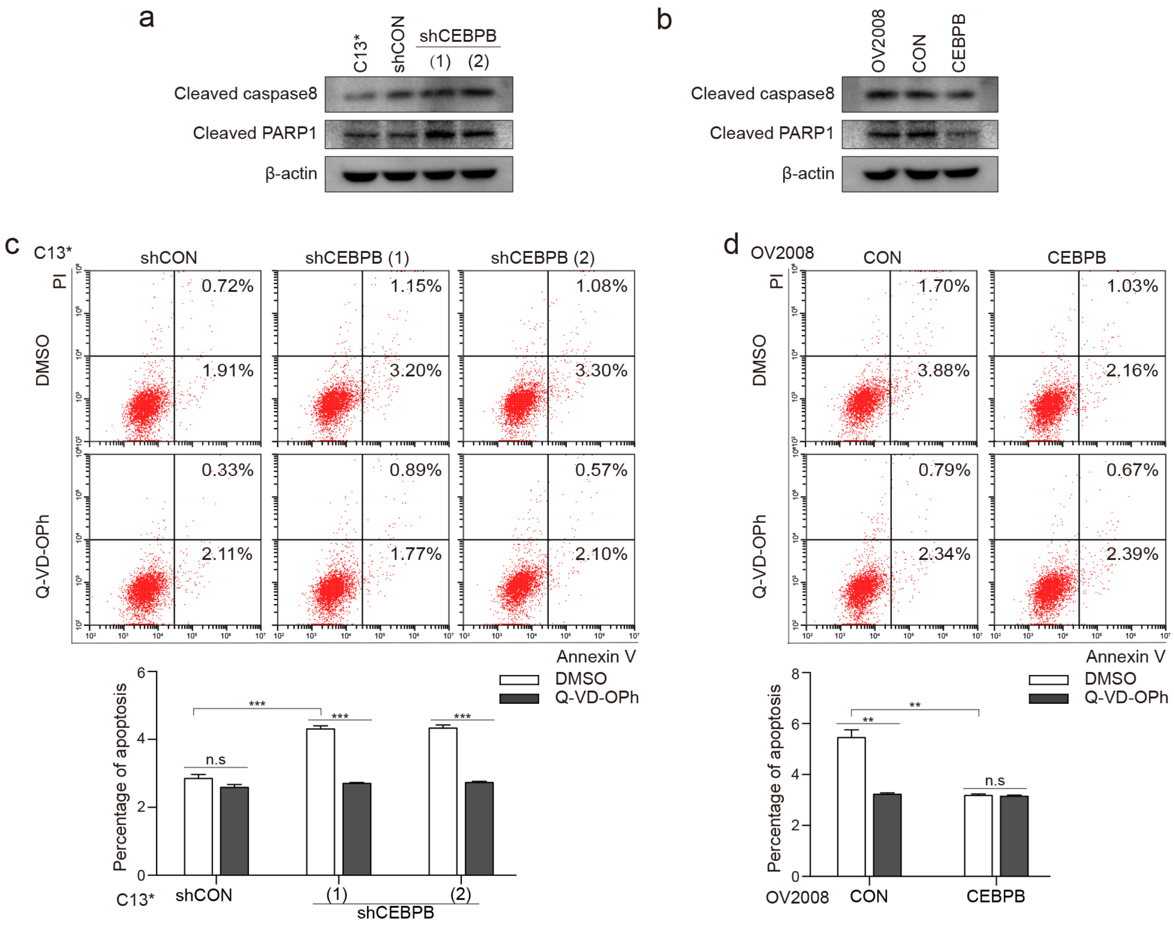

Supplementary Figure s6. C/EBPβ suppresses caspase-dependent induction of apoptosis. Western blotting analysis detected expression of cleaved caspase8 and cleaved PARP1 after C/EBPβ manipulation in C13* (a) and OV2008 (b). By western blotting analysis, all experiments were repeated three times. Apoptosis rates of C13* (c) and OV2008 (d) were measured using flow cytometry after C/EBPβ manipulation and Q-VD-OPh treatment. Each sample had triplicates and error bars were mean ± SEM of three experiments (Student's *t*-test). *P* value was denoted as * $P < 0.05$, ** $P < 0.01$, and *** $P < 0.001$, "n.s" represents "not significant".

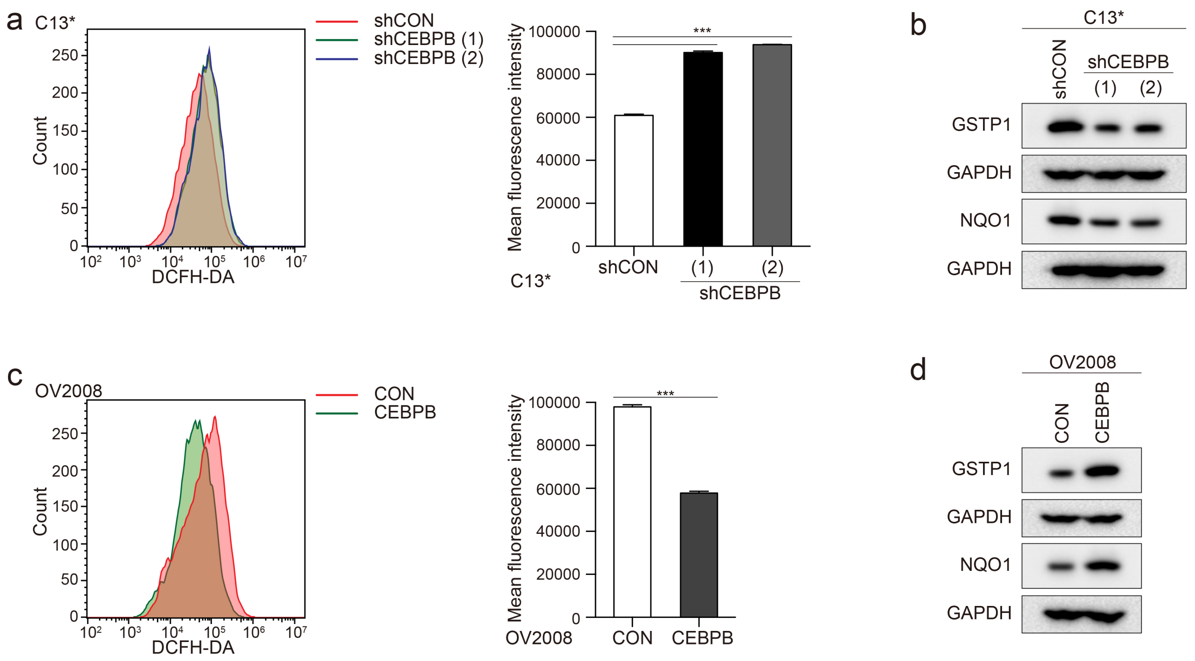

Supplementary Figure s7. C/EBPβ expression affects ROS distribution. (a) C13* shCON and shCEBPB cells were stained with DCFH-DA and analyzed for ROS distribution. Mean fluorescence intensity of DCFH-DA was shown as histogram. Each assay was conducted in triplicates and repeated three times. Data were presented as mean ± SEM (Student's *t*-test). (b) Western blotting analysis assessed expression of GSTP1 and NQO1 in C13* shCON and shCEBPB cells. The experiment was repeated thrice and representative images were shown. (c) ROS levels in OV2008 CON and CEBPB cells were detected by flow cytometry. Mean fluorescence intensity of DCFH-DA was depicted in histogram. Each assay was conducted in triplicates and error bars indicated mean ± SEM of three assays (Student's *t*-test). (d) Western blotting analysis assessed expression of GSTP1 and NQO1 in OV2008 CON and CEBPB cells and the experiments were repeated three times. *P* value was denoted as * $P < 0.05$, ** $P < 0.01$, and *** $P < 0.001$, "n.s" represents "not significant".

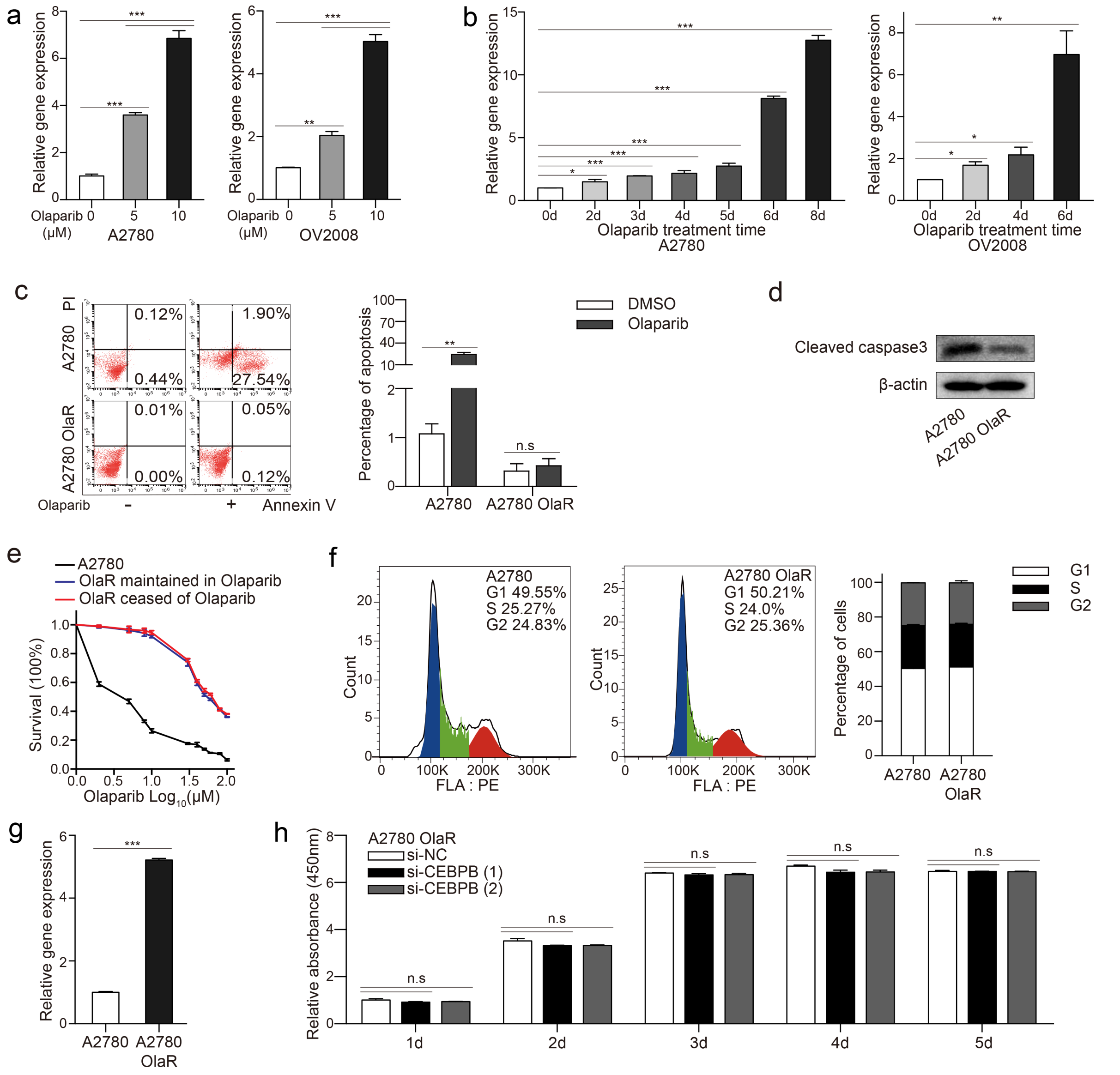

Supplementary Figure s8. Olaparib treatment increases C/EBPβ expression. A2780 and OV2008 cells were exposed to olaparib for (a) different concentrations and (b) different periods, following which CEBPB mRNA expression was quantified. RT-qPCR analyses were conducted in three wells and repeated three times. Data were presented as mean ± SEM (Student's *t*-test). (c) Apoptosis rates of A2780 and A2780 OlaR after olaparib exposure. Each assay was conducted in triplicates and error bars indicated mean ± SEM of three assays (Student's *t*-test). (d) Western blotting analysis detected cleaved caspase3 expression in A2780 and A2780 OlaR without olaparib treatment. The experiment was repeated thrice. (e) Cell

viability assays for A2780 OlaR after cessation of olaparib treatment. Each assay was conducted in six wells and repeated three times. Error bars depicted mean ± SEM. (f) Cell cycle analysis of A2780 and A2780 OlaR under olaparib exposure. Each sample had triplicate and data represented mean ± SEM of three assays. (g) Quantification of CEBPB mRNA expression in A2780 and A2780 OlaR. The experiment was repeated thrice in triplicates and error bars indicated mean ± SEM (Student's *t*-test). (h) Cell viability assays after C/EBPβ manipulation in A2780 OlaR without drug exposure. Each sample had six replicates and error bars were mean ± SEM of three experiments (Student's *t*-test). *P* value was denoted as * $P < 0.05$, ** $P < 0.01$, and *** $P < 0.001$, "n.s" represents "not significant".

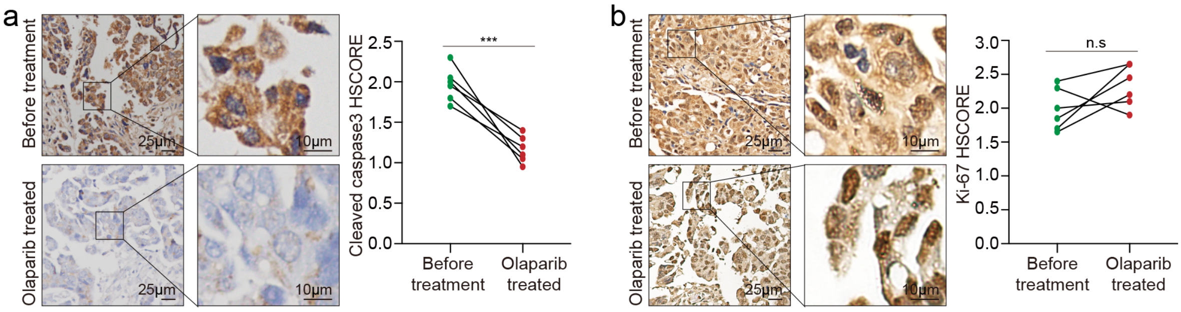

Supplementary Figure s9. Expression of cleaved caspase3 and Ki-67 in clinical specimens. Immunohistochemical analysis of (a) cleaved caspase3 and (b) Ki-67 of the clinical samples used in Fig. 3j (Student's *t*-test). *P* value was denoted as * $P < 0.05$, ** $P < 0.01$, and *** $P < 0.001$, "n.s" represents "not significant".

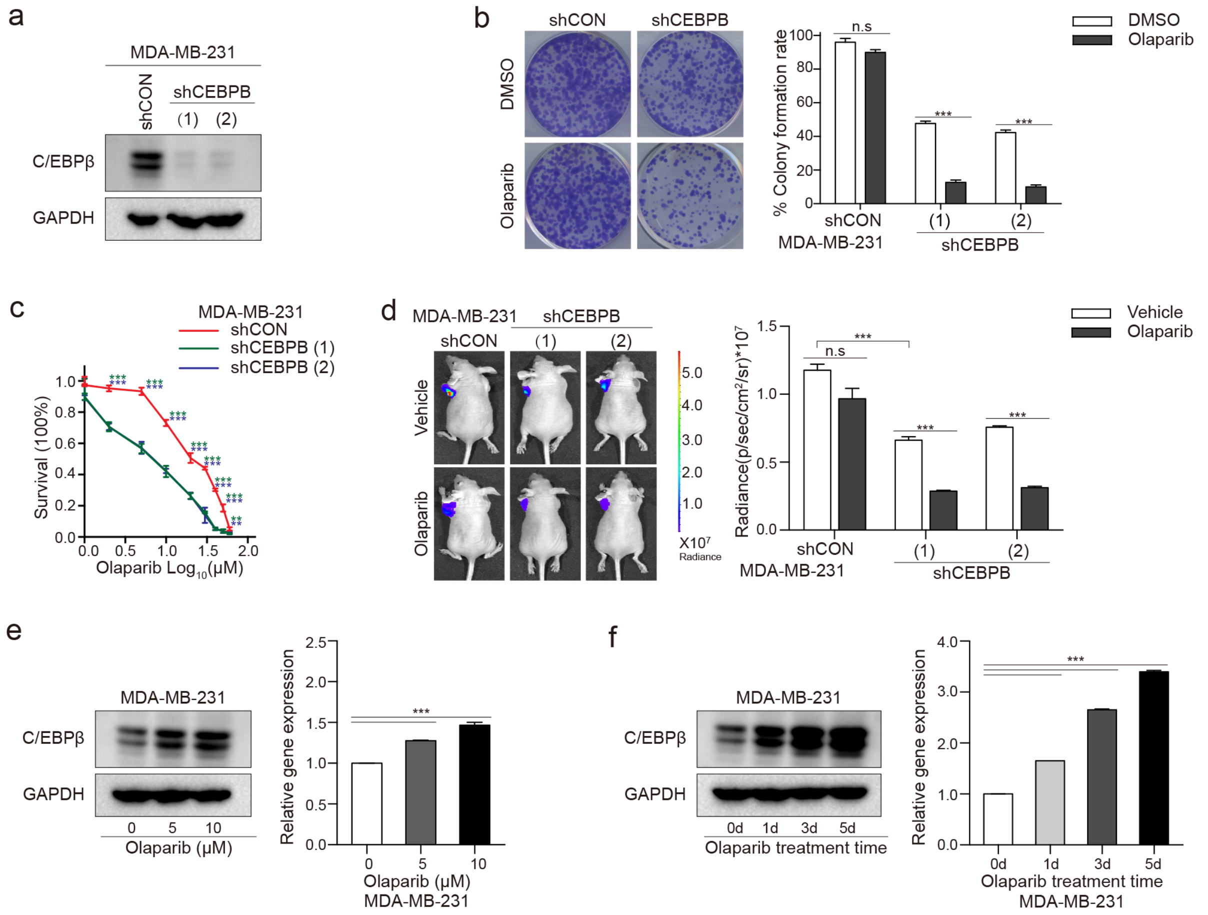

Supplementary Figure s10. The effects of C/EBPβ in MDA-MB-231. (a) Verification of C/EBPβ expression in MDA-MB-231 after C/EBPβ manipulation. Western blotting analysis was performed and repeated thrice. (b) Clonogenicity of MDA-MB-231 was detected after C/EBPβ depletion and olaparib treatment. Each assay was conducted in triplicates and error bars indicated mean ± SEM of three assays (Student's *t*-test) (c) Cell viability assays for C/EBPβ-depleted MDA-MB-231 cells. The experiment was repeated thrice in six replicates for each cell line and error bars depicted mean ± SEM (Student's *t*-test). Stably transfected MDA-MB-231 cells were subcutaneously inoculated into mouse flanks. Olaparib or vehicle was administered (six mice per group). (d) Tumor sizes were monitored using *in vivo* bioluminescent imaging, and tumor burdens were quantified by total radiance. Representative images of mouse xenografts were shown (Student's *t*-test). Error bar, mean ± SEM. MDA-MB-

231 cells were exposed to olaparib for (e) different concentrations and (f) different periods. Subsequently, C/EBPβ protein expression and CEBPB mRNA expression was measured. By western blotting analysis, all experiments were repeated three times. By RT-qPCR analysis, each sample had triplicates and error bars represented mean ± SEM of three experiments (Student's *t*-test). *P* value was denoted as * $P < 0.05$, ** $P < 0.01$, and *** $P < 0.001$, "n.s" represents "not significant".

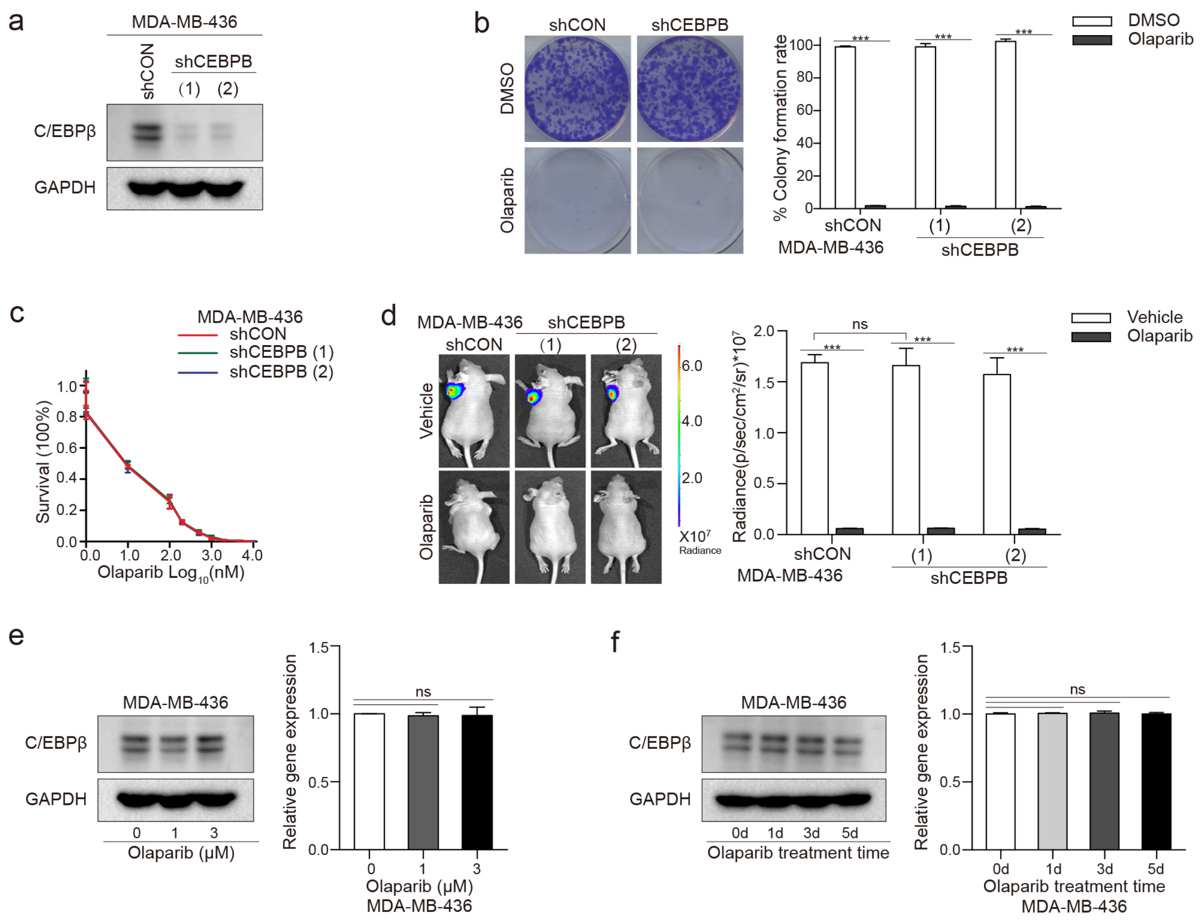

Supplementary Figure s11. The effects of C/EBPβ in MDA-MB-436. (a) Verification of C/EBPβ expression in MDA-MB-436 after C/EBPβ manipulation. Western blotting analysis was performed and repeated thrice. (b) Colony-formation rates of MDA-MB-436 were detected after C/EBPβ depletion and olaparib treatment. Each assay was conducted in triplicates and error bars indicated mean ± SEM of three assays (Student's *t*-test). (c) Cell viability assays for C/EBPβ-depleted MDA-MB-436 cells. The experiment was repeated thrice in six replicates for each cell line and error bars depicted mean ± SEM. Stably transfected MDA-MB-436 cells were subcutaneously inoculated into mouse flanks. Olaparib or vehicle was administered (six mice per group). (d) Tumor sizes were monitored using *in vivo* bioluminescent imaging, and tumor burdens were quantified by total radiance. Representative images of mouse xenografts were shown (Student's *t*-test). Error bar, mean ± SEM. MDA-MB-436 cells were exposed to

olaparib for (e) different concentrations and (f) different periods. Subsequently, C/EBPβ protein expression and CEBPB mRNA expression was measured. By western blotting analysis, all experiments were repeated three times. By RT-qPCR analysis, each sample had triplicates and error bars represented mean ± SEM of three experiments (Student's *t*-test). *P* value was denoted as * $P < 0.05$, ** $P < 0.01$, and *** $P < 0.001$, "n.s" represents "not significant".

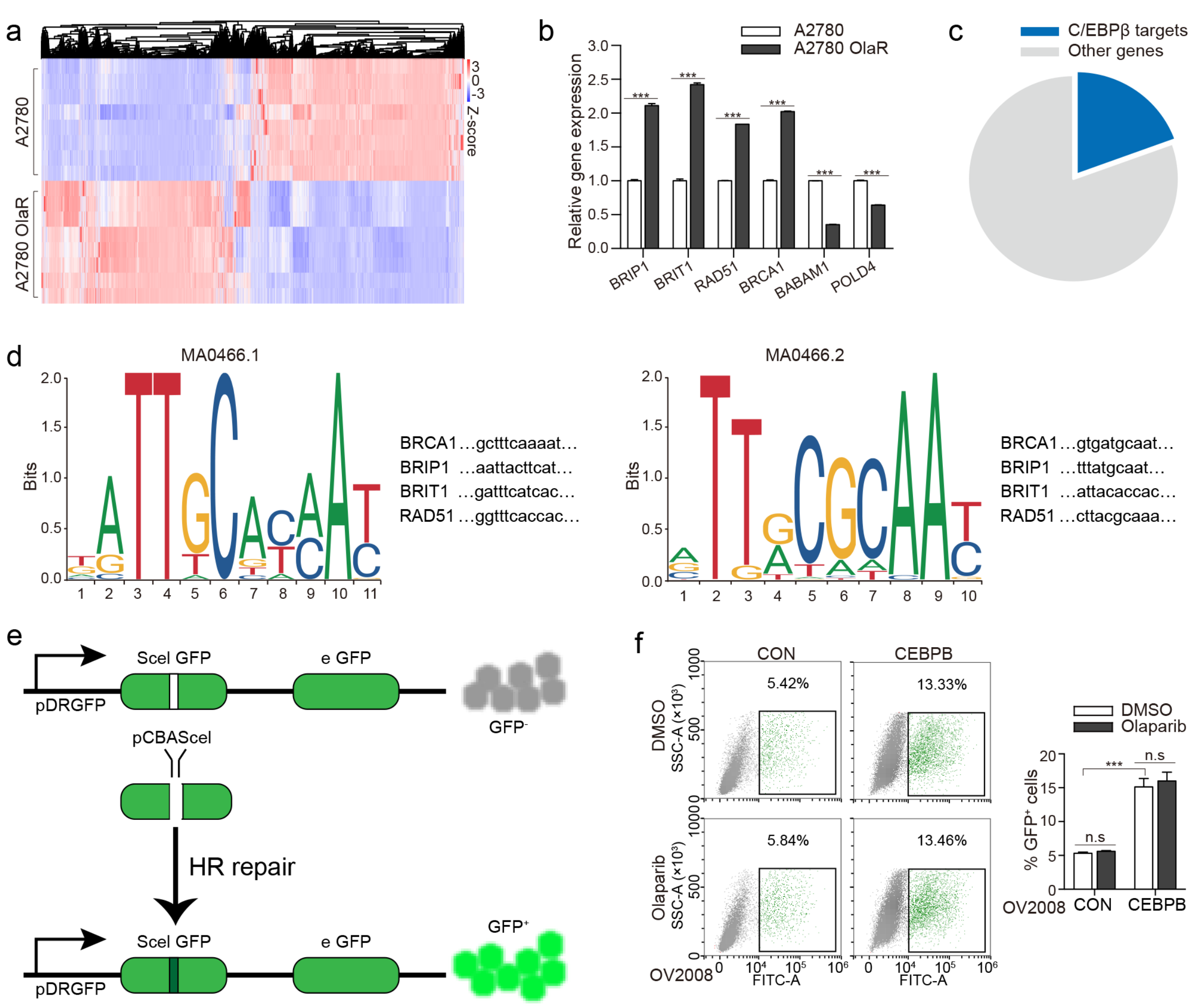

Supplementary Figure s12. C/EBPβ modulates HR genes and affects HR repair. RNA-sequencing of A2780 and A2780 OlaR was performed. (a) The DEGs identified were depicted in heatmap. (b) Results of RNA-sequencing were verified using RT-qPCR. The experiments were conducted in triplicates and repeated three times. Data were presented as mean ± SEM (Student's *t*-test) (c) Reanalysis of ChIP-seq data showed that 20% of HR genes were C/EBPβ targets. (d) Validation of C/EBPβ binding profile using JASPAR. (e) Schematic diagram of HR reporter assay. (f) HR reporter assays in OV2008 CON and CEBPB cells after olaparib treatment. Each sample had triplicates and error bars represented mean ± SEM of three experiments (Student's *t*-test). *P* value was denoted as * $P < 0.05$, ** $P < 0.01$, and *** $P < 0.001$, "n.s" represents "not significant".

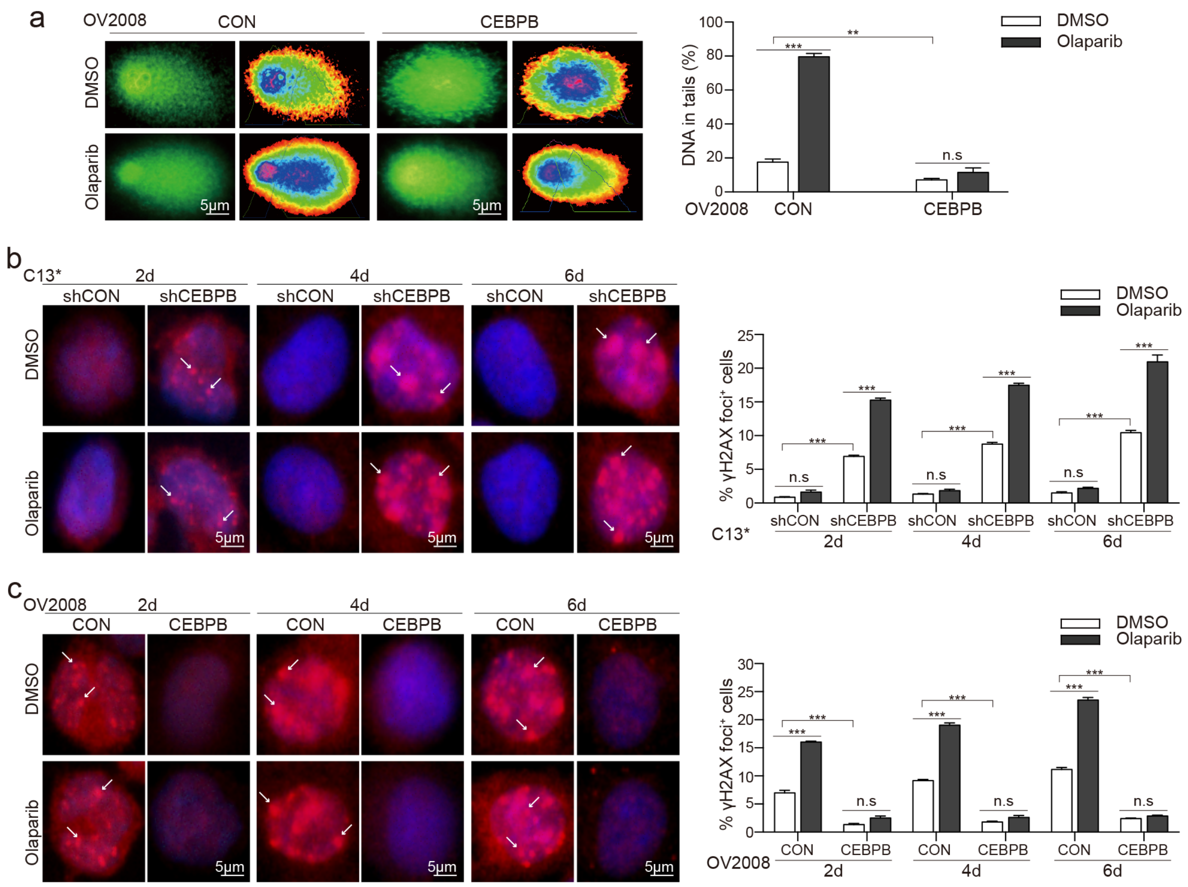

Supplementary Figure s13. C/EBPβ protects cells from DNA damage. (a) Comet assays with OV2008 CON and CEBPB cells after olaparib treatment. Results were obtained from three independent experiments and depicted as mean ± SEM (Student's *t*-test). (b) γH2AX staining in C13[*] shCON and shCEBPB cells after exposure to olaparib for different periods. Representative images were shown (left), and γH2AX foci formation rates were calculated (right). Staining foci were indicated by arrowheads. Data were obtained from three independent experiments and denoted as mean ± SEM (Student's *t*-test). (c) γH2AX staining in OV2008 CON and CEBPB cells after olaparib treatment for different periods. Representative images were shown (left), and γH2AX foci formation rates were calculated (right). Staining foci were indicated by arrowheads. Results were obtained from three independent experiments and presented as mean ± SEM (Student's *t*-test). *P* value was denoted as * $P < 0.05$, ** $P < 0.01$,

and *** $P < 0.001$, “n.s” represents “not significant”.

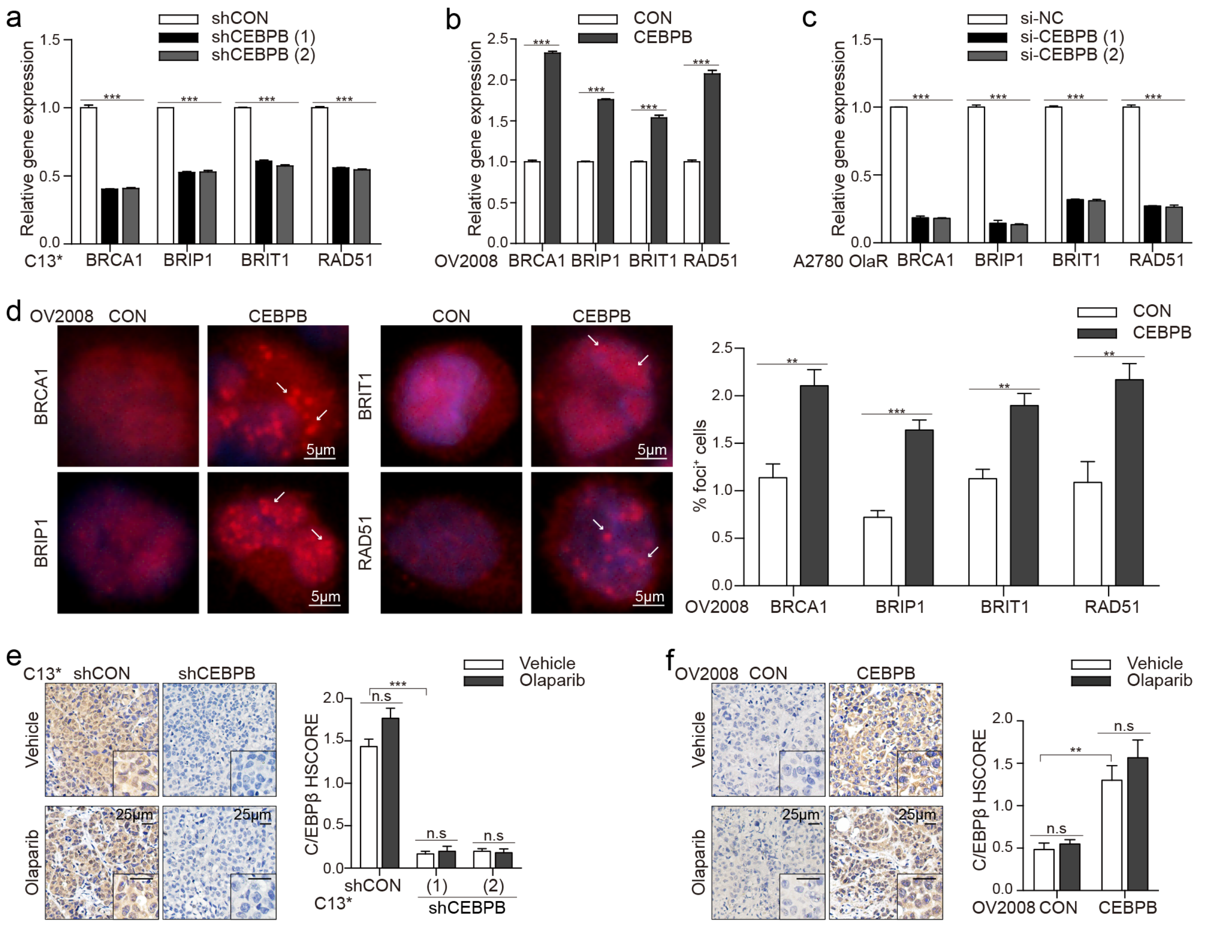

Supplementary Figure s14. C/EBPβ enhances HR genes expression. (a) mRNA expression of HR genes after C/EBPβ manipulation in C13*. (b) mRNA expression of HR genes after C/EBPβ manipulation in OV2008. (c) mRNA expression of HR genes in A2780 OlaR after siRNA interference. By RT-qPCR analysis, all experiments were conducted thrice with three replicate wells. Data were shown as mean ± SEM (Student's *t*-test). (d) HR protein foci formation rates in OV2008 CON and CEBPB cells were calculated after olaparib treatment (right). Representative images were shown (left) and staining foci were indicated by arrowheads. Results were obtained from three independent experiments and presented as mean ± SEM (Student's *t*-test). (e) C/EBPβ expression in C13* mouse models (six mice per group). Data shown represented mean ± SEM (Student's *t*-test). (f) C/EBPβ expression in OV2008 mouse models (six mice per group). Results were described as mean ± SEM (Student's *t*-test).

P value was denoted as * $P < 0.05$, ** $P < 0.01$, and *** $P < 0.001$, “n.s” represents “not significant”.

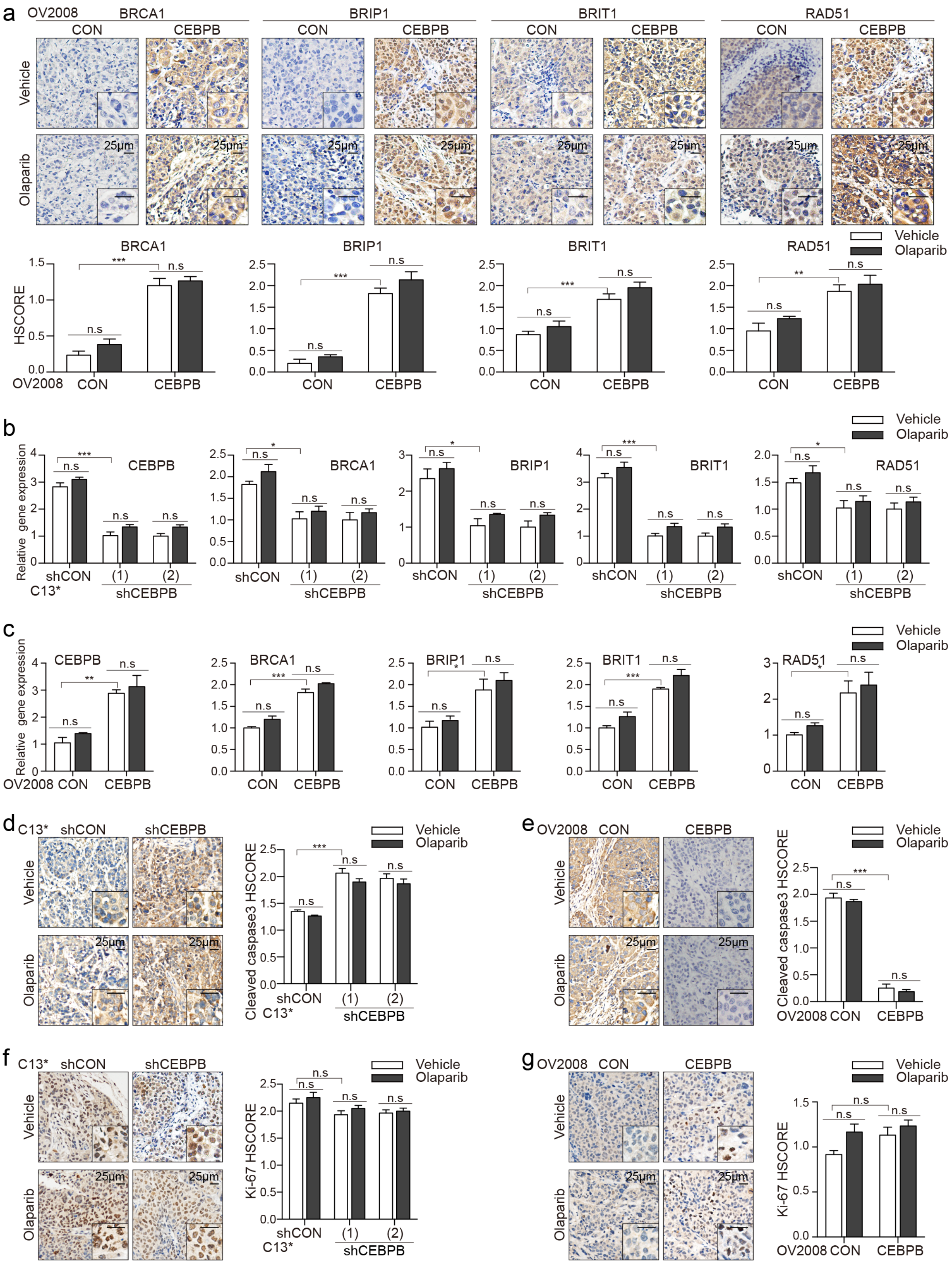

Supplementary Figure s15. C/EBPβ upregulates HR genes in *vivo*. (a) Tumor tissues from OV2008 mouse models (six mice per group) were stained with the indicated antibody, and HR proteins expression was evaluated using HSCORE. Data shown represented mean ± SEM

(Student's *t*-test) (b) mRNA expression of CEBPB and HR genes in C13* mouse models (six mice per group). (c) mRNA expression of CEBPB and HR genes in OV2008 mouse models (six mice per group). By RT-qPCR analysis, the experiment was repeated three times in triplicate wells (Student's *t*-test). Immunohistochemical analysis of cleaved caspase3 in (d) C13* and (e) OV2008 mouse models (six mice per group). Error bars denoted mean ± SEM (Student's *t*-test). Immunohistochemical analysis of Ki-67 in (f) C13* and (g) OV2008 mouse models (six mice per group). Results were described as mean ± SEM (Student's *t*-test). *P* value was denoted as * $P < 0.05$, ** $P < 0.01$, and *** $P < 0.001$, "n.s" represents "not significant".

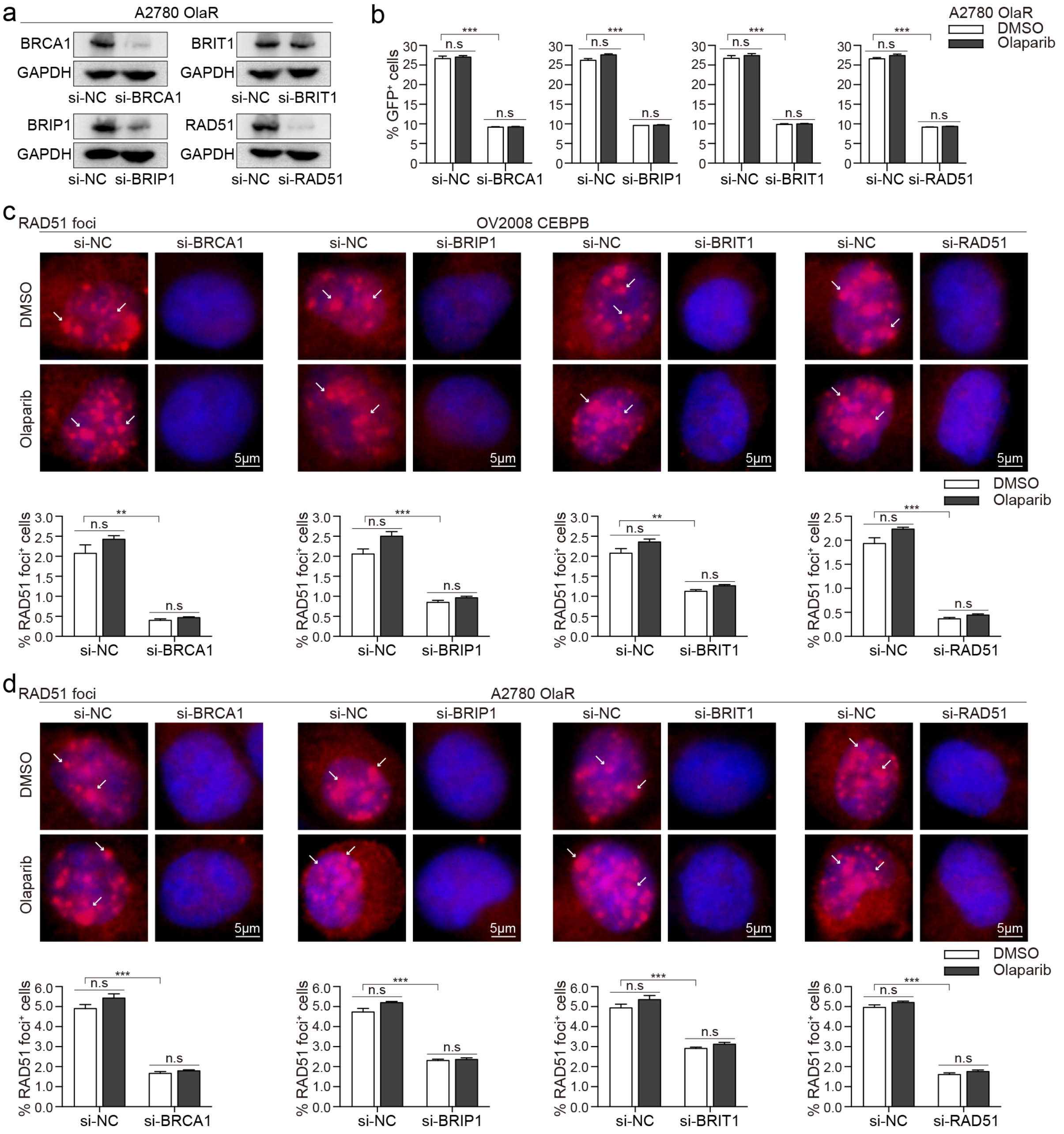

Supplementary Figure s16. siRNA interference of HR genes reduces HR capacity. (a) A2780 OlaR were transfected with siRNA targeting BRCA1, BRIP1, BRIT1, or RAD51, then HR protein expression was detected by western blot. A representative blot of three assays was shown. (b) HR reporter assays were performed in A2780 OlaR after siRNA interference and olaparib treatment. Each sample had triplicates and error bars represented mean ± SEM of three experiments (Student's *t*-test). RAD51 foci formation rates were calculated after confirmation of transfection and olaparib treatment in (c) OV2008 CEBPB and (d) A2780 OlaR (bottom).

Representative images were shown (top), and staining foci were indicated by arrowheads. Results were obtained from three independent experiments and presented as mean ± SEM (Student's *t*-test). *P* value was denoted as * $P < 0.05$, ** $P < 0.01$, and *** $P < 0.001$, "n.s" represents "not significant".

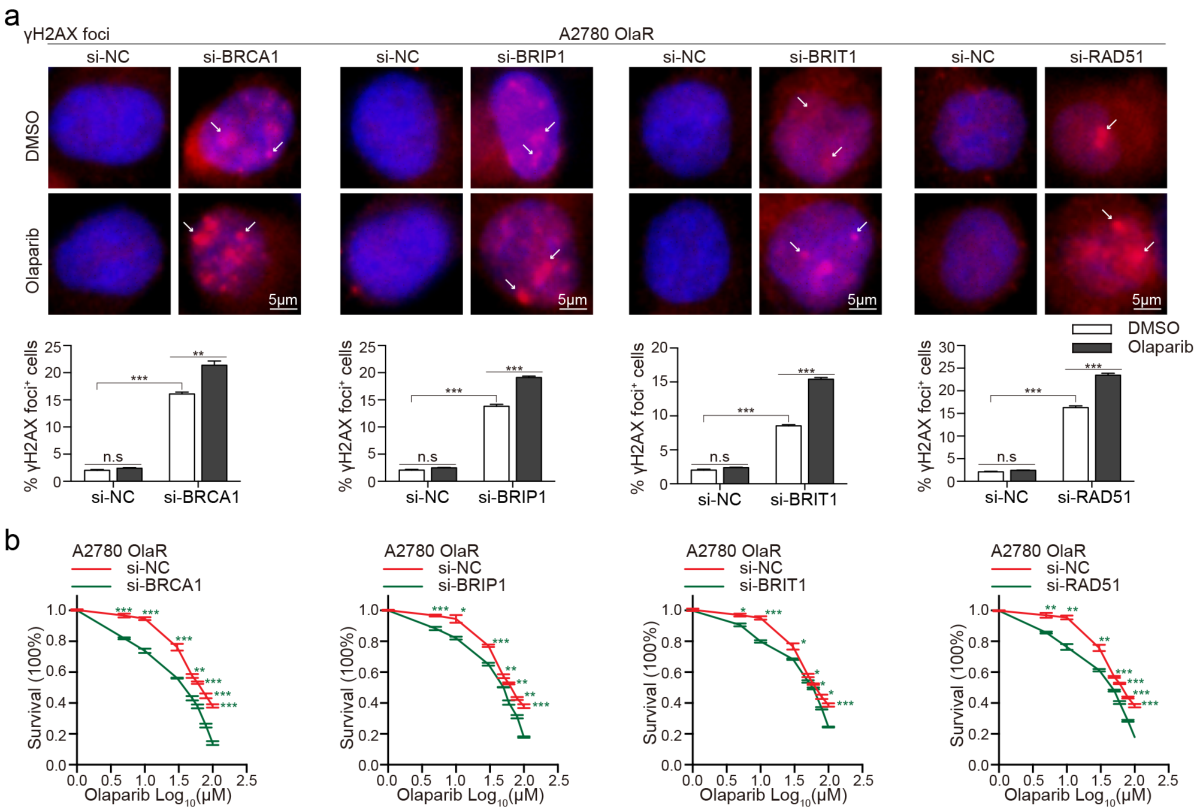

Supplementary Figure s17. siRNA interference of HR genes abrogates the effects of C/EBPβ. A2780 OlaR were transfected with siRNA targeting BRCA1, BRIP1, BRIT1, or RAD51. (a) Immunofluorescence staining of γH2AX was performed after confirmation of transfection and olaparib treatment. Representative images were shown (top), and γH2AX foci formation rates were calculated (bottom). Staining foci were indicated by arrowheads. Results were obtained from three independent experiments and presented as mean ± SEM (Student's *t*-test). (b) Cell viability assays under olaparib exposure after siRNA interference. The experiments were conducted thrice with six replicate wells. Data were shown as mean ± SEM (Student's *t*-test). P value was denoted as * $P < 0.05$, ** $P < 0.01$, and *** $P < 0.001$, "n.s" represents "not significant".
